# Social environment affects the transcriptomic response to bacteria in ant queens

**DOI:** 10.1002/ece3.4573

**Published:** 2018-10-24

**Authors:** Lumi Viljakainen, Jaana Jurvansuu, Ida Holmberg, Tobias Pamminger, Silvio Erler, Sylvia Cremer

**Affiliations:** ^1^ Ecology and Genetics Research Unit University of Oulu Oulu Finland; ^2^ School of Life Sciences University of Sussex Brighton UK; ^3^ Institute of Biology, Molecular Ecology Martin‐Luther‐University Halle‐Wittenberg Halle (Saale) Germany; ^4^ Institute of Science and Technology Austria (IST Austria) Klosterneuburg Austria

**Keywords:** Hymenoptera, immunity, RNA‐seq, social insect, transcriptomics

## Abstract

Social insects have evolved enormous capacities to collectively build nests and defend their colonies against both predators and pathogens. The latter is achieved by a combination of individual immune responses and sophisticated collective behavioral and organizational disease defenses, that is, social immunity. We investigated how the presence or absence of these social defense lines affects individual‐level immunity in ant queens after bacterial infection. To this end, we injected queens of the ant *Linepithema humile* with a mix of gram+ and gram− bacteria or a control solution, reared them either with workers or alone and analyzed their gene expression patterns at 2, 4, 8, and 12 hr post‐injection, using RNA‐seq. This allowed us to test for the effect of bacterial infection, social context, as well as the interaction between the two over the course of infection and raising of an immune response. We found that social isolation per se affected queen gene expression for metabolism genes, but not for immune genes. When infected, queens reared with and without workers up‐regulated similar numbers of innate immune genes revealing activation of Toll and Imd signaling pathways and melanization. Interestingly, however, they mostly regulated different genes along the pathways and showed a different pattern of overall gene up‐regulation or down‐regulation. Hence, we can conclude that the absence of workers does not compromise the onset of an individual immune response by the queens, but that the social environment impacts the route of the individual innate immune responses.

## 1 INTRODUCTION

Social insects (ants, bees, wasps, and termites) are nearly ubiquitously distributed and ecologically very successful due to their advanced sociality including division of labor between castes and cooperation between individuals in a colony (Bourke & Franks, 1995). Importantly, they form colonies that show reproductive division of labor between the reproductive queen(s) and the sterile workerforce, which help the queen reproduce. Social insect societies are under particular threat of pathogens and disease, because individuals in the colony usually are closely related offspring of the mother queen(s) and because the high number of individuals facilitates pathogen transmission (Schmid‐Hempel, 1998). Each individual is protected against disease by its own hygiene behavior and physiological immune system. Yet, in order to keep infections at bay, additional colony‐level defenses have evolved that consist of collectively performed hygiene behaviors and organizational defenses, together forming the “social immunity” of the colony (Cremer, Armitage, & Schmid‐Hempel, 2007; Evans & Spivak, 2010).

Social immunity is employed to protect contaminated colony members from developing infections and to inhibit disease transmission through the colony (Cremer, Pull, & Fürst, 2018). To this end, colonies of social insects perform intense nest hygiene, for example, by enriching their nest with antimicrobial material (Chapuisat, Oppliger, Magliano, & Christe, 2007; Christe, Oppliger, Bancalà, Castella, & Chapuisat, 2003; Simone, Evans, & Spivak, 2009), cleaning their nestmates from infectious particles by grooming and chemical disinfection (Hughes, Eilenberg, & Boomsma, 2002; Rosengaus, Maxmen, Coates, & Traniello, 1998; Theis, Ugelvig, Marr, & Cremer, 2015; Tragust, Mitteregger, et al., 2013), removing diseased brood from the nest (“hygienic behavior”; Rothenbuhler, 1964; Tragust, Ugelvig, Chapuisat, Heinze, & Cremer, 2013; Ugelvig, Kronauer, Schrempf, Heinze, & Cremer, 2010), and performing destructive disinfection to stop pathogen replication and to prevent disease transmission through the colony (Pull et al., 2018).

Proximately, these social immunity behaviors occur in response to pathogen exposure, and it was shown that individual infection after bacterial injection in honeybees affects the behavior of their nestmates already 6 hr after injection so that the bacteria‐injected individuals are subject to increased allogrooming and aggression (Richard, Holt, & Grozinger, 2012). This suggests that nestmates can sense immune response, and in honeybees and ants, this has recently been shown to be mediated by cuticular hydrocarbons (Hernández López, Riessberger‐Gallé, Crailsheim, & Schuehly, 2017; Pull et al., 2018)—important cues for chemical communication in insects (Howard & Blomquist, 2005). Ultimately, all social immunity measures help to keep the colony free from disease, and to—in particular—prevent disease spread to the most valuable colony members, the reproductive queens (Cremer et al., 2018).

It has been suggested that these social immune measures may interfere with the evolution as well as expression of individual immunity (Barribeau et al., 2015; Evanset al., 2006; Viljakainen et al., 2009), possibly reducing the need for individual immune responses. Most studies have focused on the analysis of the genome and immune components, where it was found that social insects have neither strongly reduced nor enlarged immune repertoires (Barribeau et al., 2015; Simola et al., 2013), and all major insect immune pathways being represented (Toll, Imd, JAK/STAT, JNK). Recent work has shown that social, colony‐level pathogen defenses affect the functionality of individual‐level immune responses. In ants and honeybees, exposure to resin, which has antibacterial properties and which these insects use as a nest building material, leads to decreased investment in physiological immune response (Borba, Klyczek, Mogen, & Spivak, 2015; Castella, Chapuisat, Moret, & Christe, 2008; Simone et al., 2009).

The physiological immune defenses in insects comprise cellular and humoral responses, the former including phagocytosis of small microorganisms and encapsulation of larger parasites and the latter composed of several signaling pathways that culminate in the production of antimicrobial peptides and other effector molecules (Ferrandon, Imler, Hetru, & Hoffmann, 2007). The core genes encoding for these immune system components are retained across several insect orders (Viljakainen, 2015). Moreover, the immune responses are interconnected with stress responses, which in insects have an immune‐enhancing effect via stress hormones releasing energy for both stress and immune responses (Adamo, 2017). This interconnection may be particularly relevant for our study where we test the effect of worker presence or absence in the context of infection, as it is known that social isolation may induce stress and interfere with disease defense abilities in insects (Boulay, Quagebeur, Godzinska, & Lenoir, 1999; Kohlmeier, Holländer, & Meunier, 2016; Koto, Mersch, Hollis, & Keller, 2015).

**Figure 1:**
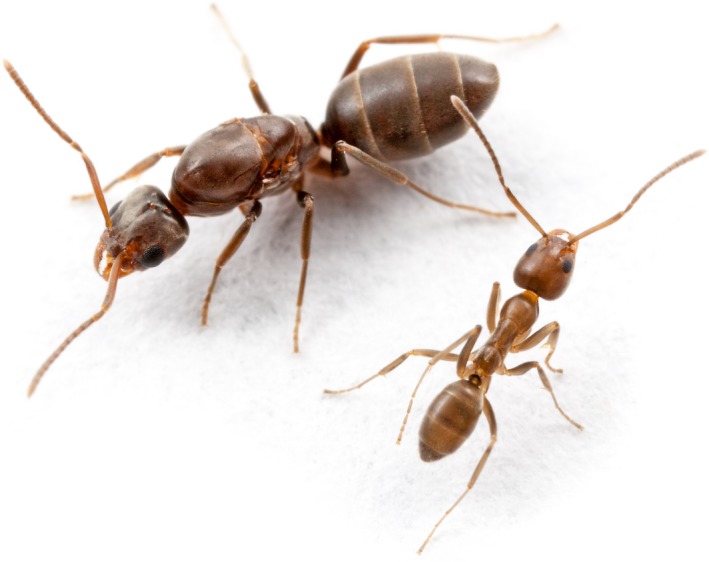
*Linepithema humile* queen (on the left) and worker. Image © Alex Wild, used by permission

While previous work has focused mostly on worker–worker interactions, we here test how ant queens, the most important individuals of the colony, modulate their individual immune defenses after bacterial infection when they have access to social immunity or not (presence or absence of workers). We control for the fact of social isolation by also determining gene expression patterns of uninfected queens when alone or in the presence of their workers. We used queens of the Argentine ant *Linepithema humile* (Figure 1) that we injected with a combination of gram+ and gram− bacteria or sterile saline solution and then kept in either isolation or with workers. After injection, changes in gene expression patterns were analyzed at four time points using RNA‐seq: shortly after injection (2 hr), during the development of the immune response (4 and 8 hr post‐injection), and when the immune response was expected to be fully activated (12 hr post‐injection) (Erler, Popp, & Lattorff, 2011). We hypothesized that both the infection state and the social environment will affect gene expression of the queens and tested for an interaction between the two, in particular if the response to bacterial infection differed between the two rearing conditions.

## 2 MATERIAL AND METHODS

### 2.1 Samples

Workers and queens of the Argentine ant *L. humile* were collected from the European main supercolony in Castell d'Aro, Spain, in April 2011 and kept in artificial nests in climate chambers (Sanyo) set to 27°C for 14 hr of light and 21°C for 10 hr of dark. The ants were fed with honey and cockroaches three times per week. Approximately 3 weeks prior to the experiments, small sub‐colonies originating from two stock colonies and each consisting of a single queen and 10 workers were placed into petri dishes (diameter 9 cm) with a plastered ground and fed with 10% sugar water.

### 2.2 Bacteria used for infections

We used the gram‐positive bacterium *Staphylococcus aureus* and the gram‐negative bacterium *Serratia marcescens* in combination for infecting the queens with the aim to induce gene expression of both Toll and Imd innate immune signaling pathways, since in *Drosophila,* gram‐positive bacteria are known to induce mainly the Toll pathway and gram‐negative bacteria the Imd pathway (Ferrandon et al., 2007). The bacteria were grown overnight in LB medium (Merck). The bacterial suspension was centrifuged, and the pellet was washed three times in sterile saline solution (hereafter called Ringer) prepared following the protocol described in Aubert & Richard (2008). The pellet from the final wash was suspended in Ringer. For the injections, bacterial suspensions were diluted, bacterial cells counted using Neubauer counting chamber, and *S. marcescens* and *S. aureus* dilutions mixed to get a solution representing both bacterial species in equal quantity.

### 2.3 Injections and social environment

*Linepithema humile* queens were studied for effects on genomewide expression patterns at four time points (2, 4, 8, and 12 hr) after bacterial versus control injections in the presence or absence of five workers in a full factorial design. All the injections were made at the same time of day (in the morning) within a time window of 3 hr. Sample information is summarized in Table 1. The queens were randomly assigned for either bacterial or Ringer injection and were first transferred to small petri dishes on ice to cold‐immobilize them for injection. Microinjections were performed using Picoliter Injector PLI‐100 Plus (Harvard Apparatus) set at 10 psi for 1 s using spiked glass needles with inner diameter of 11.9 μm (Biomedical Instruments), resulting in an injection volume of about 65 nl. This volume was injected twice between the second and third tergite of the abdomen, containing approx. 1,300 bacterial cells (50:50 mix of *S. marcescens* and *S. aureus*). The controls were injected twice with 65 nl of sterile Ringer. After injection, the queens were transferred back to their original petri dish nests and kept together with five workers (social environment, the queens referred to as “social queens” hereafter) or reared alone by removing the workers (the queens referred to as “isolated queens” hereafter) at constant room temperature (22°C) and with 10% sugar water ad libitum. Each treatment at each time point was repeated three times.

**Table 1:** Sample information

Sample ID of biological replicates[Link]	Injection	Environment	Time[Link]
B21, B37, B50	Bacteria	S	2
B5, B29, B45	Bacteria	S	4
B9, B41, B53	Bacteria	S	8
B1, B17, B33	Bacteria	S	12
B14, B22, B38	Bacteria	I	2
B6,B30, B46	Bacteria	I	4
B26, B42, B49	Bacteria	I	8
B2, B18, B55	Bacteria	I	12
C15, C39,C52	Ringer	S	2
C7, C47, C54	Ringer	S	4
C11, C27, C43	Ringer	S	8
C3, C35, C51	Ringer	S	12
C16, C24, C40	Ringer	I	2
C8, C32, C48	Ringer	I	4
C12, C28, C57	Ringer	I	8
C4, C20, C56	Ringer	I	12

I: isolated; S: social.

Underlining indicates samples excluded from the analysis due to low mapping rate.

Post‐injection time point (hours) of sample collection.

### 2.4 RNA extractions and sequencing

At 2, 4, 8, or 12 hr post‐injection (hpi), the ants were frozen in liquid nitrogen and kept in −80°C freezer until RNA extraction. The whole‐body samples were disrupted and homogenized in TissueLyser II (Qiagen) using stainless steel beads (5 mm diameter). Total RNA was extracted using RNeasy Micro Kit (Qiagen) following the protocol provided with the kit and including DNA removal using RNase‐free DNase I. RNA was quantified using Agilent 2100 Bioanalyzer, and the samples were sent to BGI Tech Solutions (Hong Kong) for library preparation (Illumina TruSeq RNA Sample Prep Kit) and mRNA sequencing (100 bp paired‐end reads) with Illumina HiSeq2000.

### 2.5 Bioinformatic analyses

The filtering of raw sequence data was performed by BGI and included adapter removal, removal of reads with more than 10% of undetermined bases, and removal of reads with more than 50% of low quality bases (*Q* < 10). Quality controlled clean data obtained from BGI were used for further analyses. The clean reads were mapped to the *L. humile* reference genome (GCF_000217595.1) using STAR v.2.4.1b (Dobin & Gingeras, 2015). The mapped reads were counted for all exons defined in the NCBI *L. humile* Annotation Release 100 (GCF_000217595.1_Lhum_UMD_V04_genomic.gff) and counts per exons were summarized for genes using HTSeq 0.9.1 (Anders, Pyl, & Huber, 2015).

The count data of the samples were visualized by principal component analysis (PCA) in R version 3.4.1 (R Core Team, 2015). Analysis of differential gene expression in each contrast at each time point was carried out using DESeq2 version 1.16.1 (Love, Huber, & Anders, 2014) in R. In DESeq2, the count data for each gene in a sample are modeled with a negative binomial distribution where the mean and dispersion are estimated from the data. The mean is the read count of a gene normalized by a size factor based on the median of the ratios of observed counts (the read count of a gene in a given sample divided by the geometric mean of the read counts of that gene across all samples), thus allowing comparison of samples with variable sequencing depth. The dispersion estimate is obtained by first estimating dispersion for each gene using maximum likelihood, then fitting a curve to the maximum likelihood estimates (MLEs), and finally, shrinking the per gene dispersion estimates toward the expected dispersion values represented by the curve using empirical Bayes’ approach. Differential expression of a given gene between two conditions of interest is analyzed by using empirical Bayes shrinkage by fitting generalized linear model (GLM) to obtain MLEs for log2 fold change (LFC) between the conditions, then fitting normal distribution (*µ* = 0) to the MLEs of all genes, and repeating the GLM fit for the given gene using this distribution as a prior. The maximum of the a posteriori distribution is the final estimate of the LFC, and the curvature of the distribution at its maximum is the standard error of the LFC. The significance of the LFCs is tested by Wald test, and the obtained *p* values are corrected for multiple testing by the method of Benjamini and Hochberg (1995). In this study, we used a false discovery rate (FDR) <10%.

Insects, including *L. humile*, are known to harbor RNA viruses (Gruber et al., 2017; Shi et al., 2016) which may have an effect on host gene expression (Doublet et al., 2017; Gerth & Hurst, 2017). We studied whether viruses are present and potentially have an effect on the gene expression patterns by assembling all the reads that could not be mapped to the *L. humile* genome by using default settings in Trinity v2.5.1, and by doing database searches with the obtained contigs against National Center for Biotechnology Information (NCBI Resource Coordinators, 2017) RefSeq virus databases “viral.1.protein.faa” and “viral.2.protein.faa” (accessed 7 January 2018) using BLASTX 2.6.0+ with an e‐value threshold of 10^−4^. Contigs that matched insect viruses and that had a query coverage of at least 400 amino acids were used in the following steps. The unmapped reads from each sample were mapped against the selected blast‐annotated virus contigs using default settings in BWA‐MEM v.0.7.17 (Li & Durbin, 2009), and the mapped reads were counted using samtools v1.4 (Li et al., 2009) and normalized with the sum of genome‐mapped and virus‐mapped reads per sample. The resulting viral load per sample was ordered by magnitude, divided into three equal sized bins, and classified as low (normalized read count range 4.27 × 10^−5^–4.45 × 10^−4^, *n* = 14), medium (normalized read count range 4.64 × 10^−4^–1.38 × 10^−3^, *n* = 16), or high (normalized read count range 2.16 × 10^−3^–0.18, *n* = 14). The virus load classification was incorporated as a factor in the analysis of differential gene expression.

A list of manually annotated immune genes, hereafter called “the core immune genes”, of the *L. humile* genome including key genes for the main signaling pathways Toll, Imd, JAK‐STAT, and JNK and additionally, genes involved in pathogen recognition, modulation of immune response, melanization, RNA interference, and clearance of microbes (antimicrobial peptides and phagocytosis; Viljakainen, 2015), was retrieved from Smith et al. (2011), Table S16. Thirteen C‐type lectins, nine scavenger receptors, two Toll‐like proteins, and transferrin were added to the list, which now totaled 121 immune genes. These immune genes were searched against the list of differentially expressed genes (DEGs). Predicted function for all the other DEGs outside the core immune genes was searched using PaperBLAST (Price & Arkin, 2017) and NCBI databases (NCBI Resource Coordinators, 2017).

A BED formatted file was parsed from the NCBI *L. humile* Annotation Release 100. The parsed BED file included the coding sequence (CDS) coordinates for genes, exon coordinates for non‐coding RNAs, and exon coordinates for some other genes without CDS annotation, excluding 210 pseudogenes and 46 tRNA genes, and merging overlapping CDS and exon regions. Based on the genomic coordinates in the BED file, the CDS or exon sequences were retrieved from the genome and concatenated for each gene in FASTA format. This resulted in total of 12,696 gene sequences. Gene Ontology (GO) annotation was carried out for the 12,696 genes using Blast2GO version 3.3.5 (Conesa et al., 2005). First, BLASTX search was done with an *E*‐value of 10^−25^ against all ant sequences in the NCBI non‐redundant database restricting the number of BLAST hits to 20. In addition, InterProScan annotation was run for the sequences (Jones et al., 2014). For the resulting hits, GO mapping and annotation were performed and InterProScan GOs were merged to annotation. The first step resulted in 9,406 sequences with GO annotations. Next, a second round of BLASTX search was done with the sequences that remained without hits in the first step with an *E*‐value of 10^−25^ against all arthropod sequences in the NCBI non‐redundant database restricting the number of BLAST hits to 100. Again, mapping and annotation were run for the resulting hits and InterProScan GOs were merged to the annotations. After the second step, 9,702 genes had GO annotations. GO term enrichment analysis was performed for all comparisons and time points to find significantly over‐ and under‐represented GO terms in the test set (DEGs, *N* > 10) with respect to the 9,702 genes with annotations as a reference set by using FatiGO package and a FDR <5% (Al‐Shahrour, Diaz‐Uriarte, & Dopazo, 2004) implemented in Blast2Go. Only the results with significant enrichment are presented in the Results.

## 3 RESULTS

**Figure 2:**
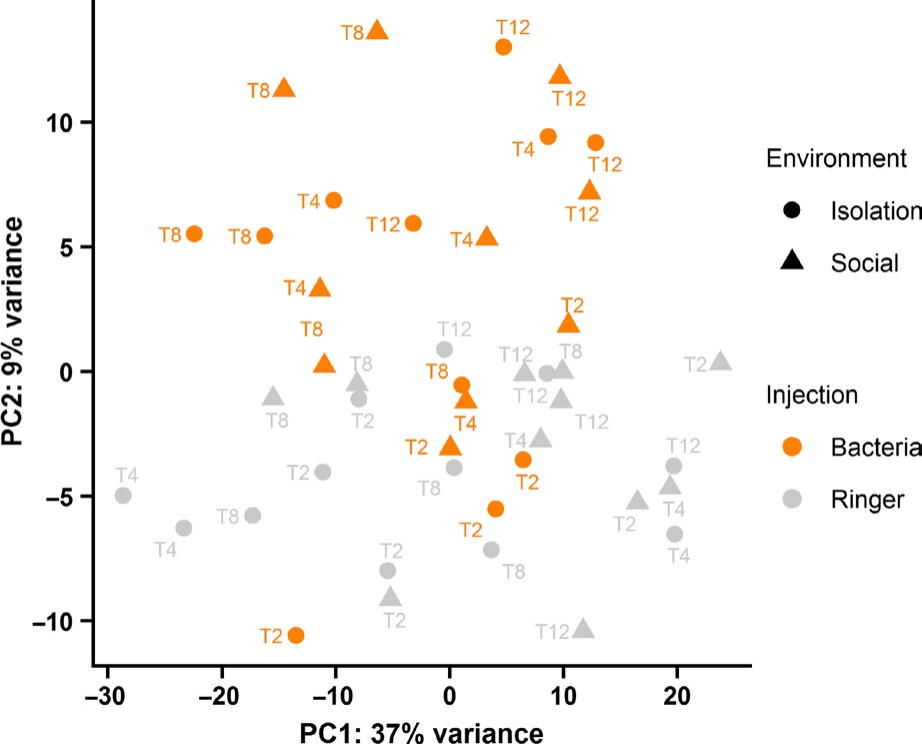
Scatterplot of the first and second principal components from principal component analysis of variance stabilized gene expression counts showing that the type of injection explains 9% of variance in gene expression

In this study, 48 transcriptomes of *L. humile* queens were sequenced (Table 1). The mean number of clean reads per sample was 32 million of which on average 93% were mapped to the *L. humile* genome and 79% mapped to the 12,952 NCBI annotated genes including protein‐coding, non‐coding, and pseudogenes (Table 2). The genome‐mapping rate was markedly lower (about 65%) for four samples: B30, B33, B50, and C54 (Tables 1 and 2), which were excluded from further analyses. The removal affected the following data points: bacteria‐injected social queens at 2 and 12 hpi, bacteria‐injected isolated queens at 4 hpi, and Ringer‐injected social queens at 4 hpi, which all involved two instead of three biological replicates. In the PCA, the first PC explaining 37% of the variation in gene expression did not separate the samples according to the treatments, but the second PC explaining 9% of variance roughly separated the samples according to the type of injection (Figure 2).

**Table 2:** RNA‐sequencing statistics

Sample	Clean reads	Mapped reads[Link]	Mapping rate (%)	Reads mapped to genes	Gene mapping rate (%)
B1	24,597,756	23,968,026	97	19,918,942	81
B14	26,624,416	25,858,946	97	21,931,220	82
B17	23,999,196	22,651,278	94	17,802,808	74
B18	23,322,168	22,207,576	95	18,369,706	79
B2	23,555,686	22,880,536	97	19,149,848	81
B21	34,493,492	33,386,034	97	28,086,624	81
B22	24,617,926	23,694,502	96	19,953,830	81
B26	29,655,470	28,194,828	95	24,070,260	81
B29	30,939,654	29,544,114	95	24,867,174	80
B30	36,209,766	24,433,280	67	20,189,926	56
B33	45,625,028	28,638,828	63	23,836,070	52
B37	44,147,540	43,202,868	98	36,453,038	83
B38	27,820,672	24,703,250	89	21,374,226	77
B41	29,808,498	27,583,454	93	23,167,504	78
B42	42,889,884	41,943,030	98	35,365,472	82
B45	36,409,122	35,492,646	97	29,598,516	81
B46	35,596,746	34,842,904	98	29,326,366	82
B49	32,376,284	31,729,918	98	27,374,740	85
B5	23,006,502	22,439,386	98	19,046,868	83
B50	27,917,926	18,466,044	66	15,435,312	55
B53	40,673,650	39,796,846	98	34,006,420	84
B55	35,647,646	34,806,466	98	29,083,614	82
B6	29,826,016	23,599,508	79	19,401,850	65
B9	23,367,018	22,771,162	97	19,052,478	82
C11	27,657,994	26,788,868	97	22,619,380	82
C12	26,274,850	25,191,632	96	21,050,254	80
C15	26,534,292	24,556,092	93	20,509,984	77
C16	22,399,506	21,708,568	97	17,984,018	80
C20	26,934,682	26,215,608	97	21,817,700	81
C24	29,162,756	27,998,298	96	23,807,766	82
C27	22,016,854	21,160,562	96	17,886,560	81
C28	20,395,710	19,525,038	96	16,514,588	81
C3	33,093,148	32,073,232	97	26,728,794	81
C32	40,544,886	37,664,904	93	32,121,400	79
C35	40,764,376	39,594,362	97	33,079,892	81
C39	38,215,078	37,412,872	98	30,819,540	81
C4	26,290,796	25,462,524	97	21,168,056	81
C40	28,242,682	27,614,780	98	23,711,616	84
C43	35,737,580	34,957,488	98	29,299,620	82
C47	44,515,826	43,220,512	97	36,959,312	83
C48	34,970,550	34,112,530	98	28,931,718	83
C51	32,295,292	31,631,658	98	26,822,394	83
C52	39,323,204	38,495,180	98	32,743,424	83
C54	44,103,734	27,761,612	63	23,261,200	53
C56	39,100,626	38,055,426	97	31,667,754	81
C57	31,438,698	30,558,738	97	26,153,756	83
C7	28,206,328	27,338,530	97	23,073,008	82
C8	21,029,550	20,324,252	97	17,309,046	82
Mean	31,507,855	29,297,056	93	24,643,825	79

Both reads of the read pair properly mapped.

### 3.1 Viral load of queens

**Figure 3:**
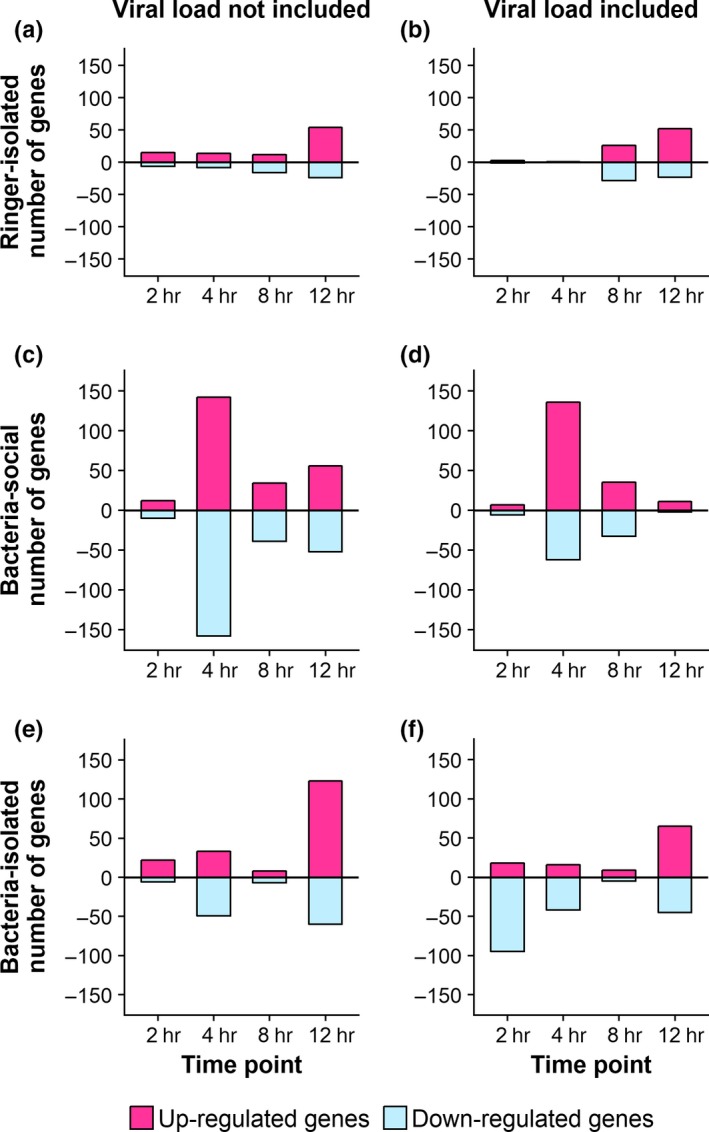
Differentially expressed genes induced by (a) isolation in Ringer‐injected control queens without viral load as a cofactor, (b) isolation in Ringer‐injected control queens and viral load taken into account as a cofactor, (c) bacterial injection in queens at social environment without viral load as a cofactor, (d) bacterial injection in queens at social environment and viral load taken into account as a cofactor, (e) bacterial injection in isolated queens without viral load as a cofactor, (f) bacterial injection in isolated queens and viral load taken into account as a cofactor

Eight RNA viruses were identified in the Trinity‐assembled contigs of reads that could not be mapped to the *L. humile* genome (Viljakainen, Holmberg, Abril, & Jurvansuu, 2018). We found that all our samples contained RNA viruses at different loads. The type of injection (bacteria or Ringer) did not affect the virus loads per sample (two‐tailed *t* test: *t* = −1.36, *df* = 22.49, *p* = 0.19), neither did the rearing condition (two‐tailed *t* test: *t* = 0.05, *df* = 41.55, *p* = 0.95), suggesting an a priori viral load of the ants, which ranged from low to high levels. Importantly, we found that the gene expression profiles of the ants were affected by viral load (Figure 3), so that we controlled for viral load in the analysis of differential gene expression.

### 3.2 Effect of social context

We first investigated the effect of social isolation per se by analyzing differentially expressed genes in Ringer‐injected queens that were either reared alone or with their workers (Figure 3, Table 3, and Appendix: Table A1). The total number of DEGs across all time points was 134 with 82 up‐regulated and 52 down‐regulated genes. GO enrichment analysis showed enrichment of biological processes “single‐organism metabolic process,” “carbohydrate phosphorylation,” and “cellular glucose homeostasis” in the up‐regulated genes at 12 hpi (Appendix: Table A2). We hence found that social context affected queen energy metabolism, but had no effect on immune gene expression.

**Table 3:** Differentially expressed genes in all treatment contrasts and time points

Contrast	Hpi	Up	Down	DEG total
BS versus CS	2	7	6	13
	4	136	62	198
	8	35	33	68
	12	11	2	13
Total		189	103	292
BI versus CI	2	18	95	113
	4	16	42	58
	8	9	5	14
	12	65	45	110
Total		108	187	295
BI versus BS	2	1	0	1
	4	0	7	7
	8	0	1	1
	12	9	2	11
Total		10	10	20
CI versus CS	2	3	1	4
	4	1	0	1
	8	26	28	54
	12	52	23	75
Total		82	52	134

B: bacteria; C: control; Hpi: Hours post‐injection; I: isolated; S: social.

### 3.3 Effect of bacterial infection depending on social context

We found that the effect of bacterial infection depended strongly on the social context the queens were reared at, even if the overall number of DEGs across all time points induced by the bacterial injection was similar across the social contexts, with 292 and 295 regulated genes in the presence and absence of workers, respectively (Figure 3, Table 3, Appendix: Tables A3 and A4). Some of the genes were regulated at several time points, and taking this into account, the number of unique DEGs was 272 in the socially reared and 268 in the isolated queens with 110 of the genes shared between the social contexts. Despite these similar numbers, the direction of regulation differed greatly: In the presence of workers, queens typically showed gene up‐regulation as compared to their Ringer control (65% of DEGs up‐regulated, χ^2^ = 25.33, *df* = 1, *p* = 4.83 × 10^−7^), whereas queens reared alone showed mostly down‐regulation (63% of DEGs down‐regulated; χ^2^ = 21.16, *df* = 1, *p* = 4.23 × 10^−6^), and only 22 of the up‐regulated genes and 27 of the down‐regulated genes were shared between the social treatments. Contrary to this overall difference in up‐regulation versus down‐regulation, both social and isolated queens showed a consistent up‐regulation of core immune genes after bacterial injection (64% up‐regulated out of 14 regulated core immune genes in social queens and 65% out of 23 in isolated queens; Table 4).

**Table 4:** Differentially expressed core immune genes induced by bacterial injection in the queens. T2, T4, T8, and T12 indicate post‐injection time points

	Locus	Annotation	Social				Isolated				
			T2	T4	T8	T12	T2		T4	T8	T12
Toll	LOC105673881	BGBP, recognition of microbes			1.18						
	LOC105673362	Serine protease, activation of the pathway				2.74	1.45				1.55
	LOC105675725	Serine protease, activation of the pathway			1.04						
	LOC105671866	Serine protease, activation of the pathway								1.31	1.70
	LOC105673363	Serine protease, activation of the pathway									1.37
	LOC105678817	Protein toll‐like		1.69			−0.94				
	LOC105678912	Protein toll‐like					−0.94				
	LOC105678648	Protein toll‐like		1.50							
	LOC105678784	Protein toll‐like									1.11
	LOC105678482	Cactus‐1a, Toll signaling							2.78		1.40
	LOC105678483	Cactus‐1b, Toll signaling									0.73
	LOC105676758	Ninjurin, up‐regulated by the Toll pathway							2.82		1.41
Imd	LOC105668729	Relish, Imd signaling					0.54				0.78
	LOC105675773	PGRP‐S2a, negative regulator of Imd pathway		−1.20					−3.24		
	LOC105678813	Pirk, negative regulator of Imd pathway									3.45
AMPs	LOC105670591	Hymenoptaecin				2.09					
	LOC105675436	Ixodidin		−1.69							
	LOC105675717	Defensin		−1.80							
Melanization	LOC105676303	Ebony		1.81							
	LOC105667585	Laccase		1.48	1.66						
	LOC105668871	Prophenoloxidase (PPO)		−2.36							−1.42
	LOC105674352	Dopa decarboxylase				1.78			3.03		
	LOC105675407	inhibin beta E chain, limits infection induced melanization								−0.77	−1.57
	LOC105677585	Serine protease, activation of PPO									1.06
	LOC105671136	Serine protease, activation of PPO									0.53
	LOC105678075	Pale, involved in melanization									1.40
	LOC105673744	serpin, inhibition of PPO activation									0.91
Phagocytosis	LOC105678691	SCR‐B‐like, phagocytosis	−2.28						−2.75		
	LOC105677178	TepII, phagocytosis							1.89	0.73	1.02
	LOC105675281	Nim‐C, mediates phagocytosis							−1.67		
	LOC105671541	Croquemort, phagocytosis									−0.68

The majority of the DEGs were not directly related to immune response, the core immune genes representing only 5% and 9% of the regulated genes in social and isolated queens, respectively. To get insight on the affected biological processes and molecular functions, GO enrichment analysis was carried out for treatment contrasts with at least 10 DEGs. The up‐regulated genes of social queens at 4 hpi showed enrichment of proteolysis and serine‐type endopeptidase inhibitor activity (Appendix: Table A2). In the isolated queens, down‐regulated genes at 2 hpi showed enrichment of proteolysis, molybdopterin cofactor biosynthetic process, and serine protease inhibitor activity (Appendix: Table A2). The genes categorized as being involved in proteolysis and having serine protease inhibitor activity were largely the same genes as in the social queens. Oxidation‐reduction process was enriched in the down‐regulated genes of isolated queens at 12 hpi including gene encoding for phenoloxidase (LOC105668871) and several cytochrome P450 protein‐coding genes.

### 3.4 Activation of Toll signaling pathway

The expression of immune genes indicated activation of the signaling pathway Toll (Table 4). In social queens, five Toll pathway genes were up‐regulated. These included *beta‐1,3‐glucan‐binding protein* (BGBP, LOC105673881), based on BLASTP search similar to *Drosophila* microbial recognition protein GNBP1 (GenBank Accession number NP_524142, 34% identity, 92% coverage) involved in recognition of gram‐positive bacteria (Pili‐Floury et al., 2004). Up‐regulated were also two genes encoding serine proteases: *limulus clotting factor C‐like* (LOC105673362) similar to *Drosophila* modular serine protease, modSP (NP_536776, 27% identity, 97% coverage), and *serine protease snake‐like* (LOC105675725) similar to *Drosophila* snake (NP_524338, 39% identity, 56% coverage). Both modSP and snake are involved in activation of the Toll receptor by transmitting microbial recognition signals from pattern‐recognition proteins GNBP1 and PGRP‐SA to Spätzle, which functions just upstream of Toll (Buchon et al., 2009). Two genes encoding Toll receptors were up‐regulated, LOC105678817 and LOC105678648, both similar to *Drosophila* Toll (NP_524518, 29% identity, 70% coverage and 30% identity, 48% coverage, respectively).

In the isolated queens, seven Toll pathway genes were up‐regulated and two down‐regulated (Table 4). Three genes encoding for serine proteases, all involved in the activation of the Toll pathway in a similar way described above for modSP and snake, were up‐regulated: two *limulus clotting factor C‐like* (LOC105673362 and LOC105673363) similar to *Drosophila* modSP (NP_536776, 27% identity, 97% coverage and 31% identity, 94% coverage, respectively) and additionally, *serine protease gd‐like* (LOC105671866) similar to gastrulation‐defective in *Drosophila* (NP_001303552, 29% identity, 84% coverage) that presumably activates serine protease snake (Rose et al., 2003). One gene encoding Toll‐like protein (LOC105678784) similar to *Drosophila* Toll (NP_524518, 36% identity, 90% coverage), which is a transmembrane receptor, was up‐regulated, and two Toll‐like protein‐coding genes were down‐regulated (LOC105678817 and LOC105678912). Two genes downstream of Toll receptor were up‐regulated, *cactus‐1a* (LOC105678482) and *cactus‐1b* (LOC105678483), both similar to *Drosophila* cactus (NP_476943, 43% identity, 50% coverage and 38% identity, 76% coverage, respectively) which is an inhibitor of NF‐κB transcription factor Dorsal that positively regulates the transcription of antimicrobial peptides (AMPs; Ferrandon et al., 2007). Notably, our analysis only revealed two Toll pathway genes overlapping between the social and isolated queens, *limulus clotting factor C‐like* (LOC105673362) and *protein Toll‐like* (LOC105678817), of which the latter was significantly up‐regulated in the social queens while down‐regulated in the isolated queens.

### 3.5 Activation of Imd signaling pathway

Genes along the Imd pathway were not as widely represented among the DEGs as the Toll pathway genes (Table 4). Down‐regulated in both social and isolated queens was a gene encoding for peptidoglycan‐recognition protein SC2 (PGRP‐SC2, LOC105675773), which is a negative regulator of the Imd pathway (Bischoff et al., 2006). In addition, isolated queens showed up‐regulation of a gene encoding for the NF‐κB‐like transcription factor Relish (LOC105668729) (Ferrandon et al., 2007) and for uncharacterized protein (LOC105678813) similar to *Drosophila* poor Imd response upon knock‐in (NP_001286686, 39% identity, 24% coverage), which, again, is a negative regulator of the Imd pathway (Kleino et al., 2008).

### 3.6 Expression of antimicrobial peptides

The Toll and Imd signaling cascades culminate in the expression of AMPs (Ferrandon et al., 2007), which are immune effectors attacking fungal and bacterial pathogens in the host. In social queens, one AMP encoding gene, *hymenoptaecin* (LOC105670591) (Casteels, Ampe, Jacobs, & Tempst, 1993), was up‐regulated at 12 hpi, and two AMP genes were down‐regulated at 4 hpi: *defensin‐2* (LOC105675717) and *chymotrypsin inhibitor‐like* (LOC105675436) similar (41% identity, 71% coverage) to ixodidin (P83516), which has been characterized in the Cattle tick *Rhipicephalus microplus* (Fogaça et al., 2006).

### 3.7 Melanization

Melanization—an active mechanism to encapsulate pathogens within the host—was induced in both social and isolated queens indicated by a number of up‐regulated genes (Table 4), for example, *venom protease‐like* (LOC105677585) similar to venom serine protease (B5U2W0, 50% identity, 96% coverage) from *Bombus ignitus*, which is shown to activate the phenoloxidase cascade (Choo et al., 2010). Once activated, phenoloxidase catalyzes the production of quinones, which are polymerized to microbicidal melanin (De Gregorio et al., 2002). Up‐regulated were also *N‐(5‐amino‐5‐carboxypentanoyl)‐l‐cysteinyl‐d‐valine synthase* (LOC105676303) similar to *ebony* from *Drosophila* (NP_524431, 47% identity, 98% coverage) and *aromatic‐l‐amino acid decarboxylase* (LOC105674352), similar to *Drosophila dopa decarboxylase* (NP_724164, 74% identity, 90% coverage), both involved in melanization (Riedel, Vorkel, & Eaton, 2011).

### 3.8 Phagocytosis

Three genes involved in phagocytosis were down‐regulated (Table 4). These involved a gene encoding for scavenger receptor class B member 1‐like (LOC105678691) and protein croquemort‐like (LOC105671541), both similar to *Drosophila* croquemort (Q27367, 28% identity, 75% coverage and 37% identity, 90% coverage, respectively) required in the uptake of bacteria by phagocytes (Guillou, Troha, Wang, Franc, & Buchon, 2016), and fibrillin‐1 (LOC105675281) similar to *Drosophila* eater (NP_651533, 37% identity, 49% coverage) that is also a phagocytic receptor promoting engulfment of bacteria (Kocks et al., 2005). Up‐regulated was a gene encoding for alpha‐2‐macroglobulin‐like protein 1 (LOC105677178) similar to *Drosophila* TepII (NP_723300, 34% identity, 37% coverage) that recognizes surface structures of bacteria leading to phagocytosis (Shokal, Kopydlowski, & Eleftherianos, 2017). Here, only the gene encoding for scavenger receptor class B member 1‐like (LOC105678691) was regulated in both social and isolated queens.

### 3.9 Interaction effect of social isolation and bacterial injection

An interaction analysis of social isolation and bacterial injection at all the four time points showed regulation of 20 genes across all time points in the bacteria‐injected isolated queens (Tables 3 and 5). These included down‐regulation at 4 hpi of the Toll receptor activator‐gene *spätzle* (LOC105678357), up‐regulation at 12 hpi of the Imd pathway signaling gene *imd* (LOC105672003) and down‐regulation, also at 12 hpi, of *hemocyte protein–glutamine gamma‐glutamyl transferase‐like* (LOC105670674) similar to *transglutaminase* (NP_609174, 37% identity, 90% coverage) in *Drosophila* that inhibits the Imd pathway transcription factor Relish (Maki, Shibata, & Kawabata, 2017).

**Table 5:** Differentially expressed genes in bacteria‐injected isolated queens in interaction analysis of social environment and bacterial treatment with false discovery rate <10%. T2, T4, T8, and T12 indicate the post‐injection time points

Time point	Locus	Description	LFC	*p*‐Value	Adjusted *p*‐value
T2	LOC105672931	Uncharacterized protein LOC105672931	1.41	5.31E−06	6.69E−02
T4	LOC105678357	Protein spätzle	−1.40	2.61E−10	3.28E−06
	LOC105668988	Uncharacterized LOC105668988	−7.82	5.81E−08	3.64E−04
	LOC105667488	Uncharacterized LOC105667488	−2.50	1.71E−05	6.36E−02
	LOC105668757	Non‐coding RNA	−1.74	2.03E−05	6.36E−02
	LOC105668733	Lambda‐crystallin homolog	−0.55	3.13E−05	6.55E−02
	LOC105678842	Acyl‐CoA Delta(11) desaturase‐like	−1.71	3.13E−05	6.55E−02
	LOC105676303	N‐(5‐amino‐5‐carboxypentanoyl)‐l‐cysteinyl‐d‐valine synthase	−1.40	5.04E−05	9.03E−02
T8	LOC105675386	Sodium‐coupled monocarboxylate transporter 1	−0.65	3.45E−06	4.35E−02
T12	LOC105677280	Uncharacterized LOC105677280	2.01	8.53E−11	9.68E−07
	LOC105674672	Cholesterol desaturase daf‐36	1.49	6.29E−08	3.57E−04
	LOC105675029	Uncharacterized LOC105675029	−0.83	1.20E−06	4.54E−03
	LOC105677088	Cytochrome P450 9e2‐like	0.99	2.24E−06	6.36E−03
	LOC105670674	Hemocyte protein–glutamine gamma‐glutamyl transferase‐like	−0.59	9.36E−06	1.77E−02
	LOC105672003	Uncharacterized (imd)	0.81	9.08E−06	1.77E−02
	LOC105673930	MLX‐interacting protein	0.47	2.47E−05	4.01E−02
	LOC105669664	Uncharacterized LOC105669664	1.17	8.56E−05	9.87E−02
	LOC105671661	Inositol polyphosphate multikinase	0.47	9.57E−05	9.87E−02
	LOC105675400	Uncharacterized LOC105675400	0.50	8.01E−05	9.87E−02
	LOC105676429	Protein embryonic gonad‐like	0.73	9.19E−05	9.87E−02

LFC: log_2_ fold change.

Hence, in both presence and absence of workers, all important immune defense pathways were triggered in the queens, with the highest number in the Toll pathway.

## 4 DISCUSSION

In this study, we tested how the presence or absence of workers affects ant queen immune response after bacterial infection. When testing for the effect of our experimentally induced bacterial infections, we found that existing viral load had an effect on differential gene expression analyses and that virus presence and load should be taken into account in these types of analysis, as previously reported (Gerth & Hurst, 2017). To further control for the effect of social worker presence or absence per se in the absence of an infection, we first analyzed differential gene expression between Ringer‐injected queens reared in the social environment or alone. Despite our sample size being large enough to detect significant effects of rearing on queen metabolism, we could not detect any general effect on queen immune gene expression.

We then tested whether worker presence or absence interfered with the queens’ individual immune response to bacterial infection over the course of infection. Overall, we found that injection of bacteria, over the four time points studied, affected the expression of similar numbers of genes in both social and isolated queens but interestingly, the social queens up‐regulated the majority of the genes, whereas in the isolated queens, down‐regulation was prevailing. This general down‐regulation might be a consequence of social isolation, which has been shown to affect life‐history traits by reducing longevity in workers of the ant *Camponotus fellah* (Boulay et al., 1999; Koto et al., 2015), yet did not compromise the innate immune response of bumblebees after pathogen challenge (Richter, Helbing, Erler, & Lattorff, 2012). In the group‐living earwig *Forficula auricularia*, rearing individuals alone also lead to a transiently increased susceptibility after pathogen exposure shortly after isolation, yet an indistinguishable survival of individuals living isolated or in groups for longer periods (Kohlmeier et al., 2016), hence the effects of social isolation may be plastic, both varying over time and across species.

The isolated queens regulated slightly, but not significantly, higher number of the core immune genes than social queens (23 vs. 14, χ^2^ = 3.38, *df* = 1, *p* = 0.07) and the immune gene expression in the isolated queens did not show the overall pattern of down‐regulation observed in all of their DEGs: 65% of immune genes were up‐regulated as opposed to 37% of all DEGs (χ^2^ = 8.11, *df* = 1, *p* = 0.004). Therefore, even though gene expression in the isolated queens showed a trend of down‐regulation, the activation of cellular and humoral immune cascades was comparable to the social queens.

An interesting observation was two enriched GO terms, serine protease inhibitor activity and proteolysis, both categories including approximately 20 genes, which were up‐regulated at 4 hpi in the bacteria‐injected social queens and down‐regulated at 2 hpi in the bacteria‐injected isolated queens. All except one of the genes in the serine protease inhibitor‐category were long non‐coding RNAs (lncRNA) which Blast2Go annotation found to contain a protease inhibitor domain suggesting they might regulate serine protease inhibitors (serpins). In insects, serpins are known to be involved in the regulation of immune signaling cascades, phagocytosis, and digestion (Gubb, Sanz‐Parra, Barcena, Troxler, & Fullaondo, 2010), and the expression of serpin‐related lncRNAs could be involved in the fine‐tuning of various arms of immune response. It is striking that the gene expression patterns of these genes showed opposite directions in the two rearing conditions, highlighting the strong effect of social environment on the general response to bacterial injection.

As a conclusion, this study shows that ant queens were equally able to activate innate immune signaling cascades after bacterial injection when kept together with workers or when reared alone. This reveals that pathogen‐injected queens raise an induced immune response even in the presence of rearing workers, yet that worker presence interferes with which exact set of genes is regulated. Hence, we could show that individual queen responses are not compromised, but modulated by their social context.

## AUTHOR CONTRIBUTIONS

LV and SC designed the research, LV performed the experimental work, LV carried out the bioinformatics analyses of RNA‐seq data, LV and JJ carried out the virus‐related bioinformatics analyses, LV and IH performed the statistical analyses, TB and SE helped LV to interpret the results related to differentially expressed genes, and LV and SC wrote the manuscript with contributions from all authors.

## DATA ACCESSIBILITY

The short read sequence data are deposited at NCBI, BioProject PRJNA279338.

## APPENDIX 1

**Table A1:** Differentially expressed genes in Ringer‐injected isolated queens with false discovery rate <10%. T2, T4, T8, and T12 indicate the post‐injection time points

Time point	Locus	Description	LFC	*p*‐Value	Adjusted *p*‐value
T2	LOC105680010	Uncharacterized LOC105680010 (LOC105680010)	7.62	5.41E−06	2.25E−02
	LOC105671203	Uncharacterized LOC105671203 (LOC105671203)	4.28	9.72E−13	1.21E−08
	LOC105675063	Alkaline phosphatase 4‐like (LOC105675063)	3.35	1.55E−07	9.66E−04
	LOC105670077	DNA repair protein RAD50‐like (LOC105670077)	−20.67	1.56E−05	4.87E−02
T4	LOC105668186	Uncharacterized LOC105668186 (LOC105668186)	7.47	3.34E−07	4.14E−03
T8	LOC105668186	Uncharacterized LOC105668186 (LOC105668186)	8.89	4.74E−04	9.11E−02
	LOC105670097	Spidroin‐1 (LOC105670097)	6.86	1.91E−06	1.34E−03
	LOC105678335	Gephyrin‐like (LOC105678335)	2.94	7.95E−05	3.06E−02
	LOC105677347	Uncharacterized LOC105677347 (LOC105677347)	2.34	2.36E−11	7.87E−08
	LOC105677332	Uncharacterized LOC105677332 (LOC105677332)	2.17	1.20E−07	1.99E−04
	LOC105678672		1.82	2.49E−05	1.13E−02
	LOC105671207	A disintegrin and metalloproteinase with thrombospondin motifs 7‐like (LOC105671207)	1.66	1.08E−04	4.00E−02
	LOC105677333	Uncharacterized LOC105677333 (LOC105677333)	1.65	2.00E−06	1.34E−03
	LOC105679161	A disintegrin and metalloproteinase with thrombospondin motifs 17‐like (LOC105679161)	1.61	2.17E−04	6.99E−02
	LOC105678664		1.57	2.57E−06	1.61E−03
	LOC105674354	Tyrosine aminotransferase (LOC105674354)	1.47	3.40E−04	7.94E−02
	LOC105667964	THAP domain‐containing protein 4‐like (LOC105667964)	1.27	5.27E−04	9.77E−02
	LOC105677348	Uncharacterized LOC105677348 (LOC105677348)	1.17	9.58E−07	7.80E−04
	LOC105673216	Putative fatty acyl‐CoA reductase CG5065 (LOC105673216)	1.15	3.47E−04	7.94E−02
	LOC105679050	Transcription termination factor 2‐like (LOC105679050)	1.12	2.35E−05	1.12E−02
	LOC105671872	Vitellogenin‐2‐like (LOC105671872)	0.98	1.88E−07	2.35E−04
	LOC105675620	Aminopeptidase N‐like (LOC105675620)	0.96	4.00E−04	8.24E−02
	LOC105670430	Aminopeptidase N‐like (LOC105670430)	0.90	3.45E−04	7.94E−02
	LOC105667719	Uncharacterized protein PFB0145c‐like (LOC105667719)	0.87	1.13E−05	5.97E−03
	LOC105668487	Uncharacterized LOC105668487 (LOC105668487)	0.85	2.60E−04	7.42E−02
	LOC105678648	Protein toll‐like (LOC105678648)	0.84	2.11E−05	1.06E−02
	LOC105672608	Facilitated trehalose transporter Tret1‐like (LOC105672608)	0.84	4.20E−04	8.24E−02
	LOC105669158	Sorbitol dehydrogenase‐like (LOC105669158)	0.73	2.90E−04	7.60E−02
	LOC105674664	Cytochrome P450 6a2‐like (LOC105674664)	0.71	2.59E−04	7.42E−02
	LOC105670239	Uncharacterized LOC105670239 (LOC105670239)	0.67	2.52E−04	7.42E−02
	LOC105671841	Pleckstrin homology domain‐containing family M member 2 (LOC105671841)	0.66	2.85E−04	7.60E−02
	LOC105676405	Fatty acid synthase (LOC105676405)	−0.64	1.67E−04	5.58E−02
	LOC105678111	Uncharacterized LOC105678111 (LOC105678111)	−0.64	1.41E−04	4.87E−02
	LOC105675000	Cartilage oligomeric matrix protein (LOC105675000)	−0.66	2.23E−04	6.99E−02
	LOC105676540	Uncharacterized LOC105676540 (LOC105676540)	−0.78	4.14E−04	8.24E−02
	LOC105674426	Beta‐1	−0.79	3.03E−04	7.60E−02
	LOC105675528	Angiotensin‐converting enzyme (LOC105675528)	−0.81	3.89E−04	8.24E−02
	LOC105674352	Aromatic‐l‐amino acid decarboxylase (LOC105674352)	−0.95	4.96E−04	9.37E−02
	LOC105677051	Protein Malvolio (LOC105677051)	−0.95	4.00E−04	8.24E−02
	LOC105670059	Probable serine/threonine‐protein kinase kinX (LOC105670059)	−0.96	5.60E−05	2.44E−02
	LOC105672810	Protein yellow (LOC105672810)	−1.02	7.95E−05	3.06E−02
	LOC105678067	Facilitated trehalose transporter Tret1‐like (LOC105678067)	−1.05	7.31E−05	3.05E−02
	LOC105677852	Uncharacterized LOC105677852 (LOC105677852)	−1.05	3.56E−06	2.10E−03
	LOC105677615	Alpha‐tocopherol transfer protein‐like (LOC105677615)	−1.08	3.51E−04	7.94E−02
	LOC105674532	ETS homologous factor‐like (LOC105674532)	−1.10	3.64E−08	9.11E−05
	LOC105677500	Glucosylceramidase‐like (LOC105677500)	−1.15	2.75E−04	7.60E−02
	LOC105671381	Uncharacterized LOC105671381 (LOC105671381)	−1.16	8.44E−06	4.69E−03
	LOC105668572	Uncharacterized LOC105668572 (LOC105668572)	−1.18	2.42E−07	2.69E−04
	LOC105673352	Sodium‐coupled neutral amino acid transporter 9‐like (LOC105673352)	−1.26	3.04E−04	7.60E−02
	LOC105667677	Zinc carboxypeptidase‐like (LOC105667677)	−1.27	1.52E−07	2.17E−04
	LOC105676197	5‐aminolevulinate synthase	−1.34	5.32E−08	1.06E−04
	LOC105670776	Endocuticle structural glycoprotein SgAbd‐4‐like (LOC105670776)	−1.34	1.35E−04	4.82E−02
	LOC105670441	Uncharacterized LOC105670441 (LOC105670441)	−1.43	7.43E−07	7.44E−04
	LOC105670071	Glucosylceramidase‐like (LOC105670071)	−1.45	4.18E−04	8.24E−02
	LOC105679194	Muscle segmentation homeobox‐like (LOC105679194)	−1.82	2.81E−12	1.40E−08
	LOC105668025	Transmembrane protease serine 9‐like (LOC105668025)	−2.01	3.57E−04	7.94E−02
	LOC105676839	l‐lactate dehydrogenase‐like (LOC105676839)	−2.06	1.01E−06	7.80E−04
	LOC105679457	Uncharacterized LOC105679457 (LOC105679457)	−6.72	9.72E−07	7.80E−04
	LOC105670077	DNA repair protein RAD50‐like (LOC105670077)	−7.90	1.99E−12	1.40E−08
T12	LOC105668565	Uncharacterized LOC105668565 (LOC105668565)	6.55	1.98E−04	3.56E−02
	LOC105669318	Kynurenine/alpha‐aminoadipate aminotransferase	4.72	9.56E−05	2.29E−02
	LOC105671889	Synaptobrevin homolog YKT6‐like (LOC105671889)	3.73	2.30E−05	7.35E−03
	LOC105672897		3.37	1.56E−14	3.29E−11
	LOC105679180	Solute carrier family 2	3.22	6.66E−15	1.75E−11
	LOC105669922	Uncharacterized LOC105669922 (LOC105669922)	3.05	4.44E−07	3.60E−04
	LOC105674428	Uncharacterized LOC105674428 (LOC105674428)	2.83	1.64E−12	2.19E−09
	LOC105672083	Cytochrome P450 4c21‐like (LOC105672083)	2.71	1.01E−04	2.33E−02
	LOC105674424		2.54	4.95E−15	1.74E−11
	LOC105670688	Uncharacterized LOC105670688 (LOC105670688)	2.53	1.17E−05	4.57E−03
	LOC105678416	Pancreatic lipase‐related protein 2‐like (LOC105678416)	2.44	7.24E−07	4.77E−04
	LOC105672219	Leucine‐rich repeat‐containing protein 4‐like (LOC105672219)	2.41	1.32E−04	2.67E−02
	LOC105672698	Uncharacterized LOC105672698 (LOC105672698)	2.36	6.73E−06	3.08E−03
	LOC105667310		2.34	5.18E−07	3.81E−04
	LOC105674425	Uncharacterized LOC105674425 (LOC105674425)	2.28	1.81E−04	3.35E−02
	LOC105668386	Trifunctional purine biosynthetic protein adenosine‐3 (LOC105668386)	2.24	9.29E−05	2.27E−02
	LOC105671469	d‐arabinitol dehydrogenase 1‐like (LOC105671469)	2.22	1.99E−04	3.56E−02
	LOC105673823	Glucose dehydrogenase [FAD	2.16	4.39E−06	2.31E−03
	LOC105668392	Succinate dehydrogenase [ubiquinone] iron‐sulfur subunit	2.16	1.30E−04	2.67E−02
	LOC105669349	Uncharacterized LOC105669349 (LOC105669349)	2.15	1.24E−04	2.60E−02
	LOC105669805	Facilitated trehalose transporter Tret1‐2 homolog (LOC105669805)	2.08	5.43E−04	8.40E−02
	LOC105667792	Synaptotagmin‐6 (LOC105667792)	2.00	1.08E−04	2.33E−02
	LOC105676997	Patatin‐like phospholipase domain‐containing protein 3 (LOC105676997)	1.97	3.37E−09	3.22E−06
	LOC105667804	Uncharacterized family 31 glucosidase KIAA1161 (LOC105667804)	1.92	5.44E−07	3.81E−04
	LOC105674420	3'(2')	1.89	2.22E−05	7.30E−03
	LOC105670888	Hexokinase type 2‐like (LOC105670888)	1.88	2.23E−10	2.61E−07
	LOC105678328	T‐lymphocyte activation antigen CD86‐like (LOC105678328)	1.85	6.15E−05	1.59E−02
	LOC105674418	Beta‐1	1.78	8.76E−07	5.43E−04
	LOC105668703	Membrane metalloendopeptidase‐like 1 (LOC105668703)	1.71	1.61E−05	5.70E−03
	LOC105668747	C‐1‐tetrahydrofolate synthase	1.70	2.05E−04	3.59E−02
	LOC105674681	Cytochrome P450 6j1‐like (LOC105674681)	1.63	1.68E−04	3.21E−02
	LOC105677243	Uncharacterized LOC105677243 (LOC105677243)	1.53	1.67E−04	3.21E−02
	LOC105676329	Insulin‐like growth factor‐binding protein complex acid labile subunit (LOC105676329)	1.53	5.78E−06	2.77E−03
	LOC105674747	Cytosolic purine 5'‐nucleotidase (LOC105674747)	1.51	2.64E−06	1.46E−03
	LOC105680118	Cysteine proteinase 1‐like (LOC105680118)	1.49	5.94E−04	8.74E−02
	LOC105675420	Protein yellow‐like (LOC105675420)	1.48	5.08E−05	1.37E−02
	LOC105669199	6‐phosphofructo‐2‐kinase/fructose‐2	1.43	1.35E−05	5.09E−03
	LOC105674352	Aromatic‐l‐amino acid decarboxylase (LOC105674352)	1.37	1.03E−05	4.32E−03
	LOC105667680	Probable hydroxyacid‐oxoacid transhydrogenase	1.37	1.59E−04	3.16E−02
	LOC105675723	Serine protease snake‐like (LOC105675723)	1.36	1.62E−05	5.70E−03
	LOC105668704	Organic cation transporter protein (LOC105668704)	1.36	4.04E−05	1.15E−02
	LOC105674248	Branched‐chain‐amino acid aminotransferase	1.35	2.78E−05	8.59E−03
	LOC105680007	Protein toll (LOC105680007)	1.35	2.01E−06	1.18E−03
	LOC105668437	CCAAT/enhancer‐binding protein (LOC105668437)	1.33	1.81E−04	3.35E−02
	LOC105675016	Peroxidase‐like (LOC105675016)	1.27	7.08E−04	9.94E−02
	LOC105677240	General odorant‐binding protein 56d‐like (LOC105677240)	1.18	1.06E−04	2.33E−02
	LOC105677283	Alpha‐aminoadipic semialdehyde synthase	1.14	4.72E−04	7.52E−02
	LOC105671238	Exosome component 10 (LOC105671238)	1.14	6.13E−04	8.85E−02
	LOC105675407	Inhibin beta E chain (LOC105675407)	1.10	3.55E−04	5.92E−02
	LOC105667297	Protein LTV1 homolog (LOC105667297)	1.08	4.42E−04	7.27E−02
	LOC105671942	Hexokinase‐2‐like (LOC105671942)	1.08	8.18E−05	2.05E−02
	LOC105677187	Serine protease easter‐like (LOC105677187)	1.05	1.09E−04	2.33E−02
	LOC105677615	Alpha‐tocopherol transfer protein‐like (LOC105677615)	−1.38	3.07E−05	9.22E−03
	LOC105675717	Defensing‐2 (LOC105675717)	−1.39	4.68E−04	7.52E−02
	LOC105679818	Cytosolic 10‐formyltetrahydrofolate dehydrogenase (LOC105679818)	−1.47	5.42E−04	8.40E−02
	LOC105672088	Lipid storage droplets surface‐binding protein 2‐like (LOC105672088)	−1.65	5.61E−04	8.55E−02
	LOC105677041	Cytochrome P450 4C1‐like (LOC105677041)	−1.65	6.99E−04	9.94E−02
	LOC105671746	Probable cytochrome P450 304a1 (LOC105671746)	−1.67	6.10E−08	5.35E−05
	LOC105667919	Protein crumbs‐like (LOC105667919)	−1.72	5.98E−04	8.74E−02
	LOC105677480	Uncharacterized LOC105677480 (LOC105677480)	−1.73	5.26E−06	2.64E−03
	LOC105668144	1‐acyl‐sn‐glycerol‐3‐phosphate acyltransferase alpha‐like (LOC105668144)	−1.82	8.58E−06	3.76E−03
	LOC105677809	Uncharacterized LOC105677809 (LOC105677809)	−1.86	1.81E−05	6.14E−03
	LOC105674506	Annulin (LOC105674506)	−1.92	1.09E−05	4.43E−03
	LOC105675090	Uncharacterized LOC105675090 (LOC105675090)	−1.94	4.21E−05	1.17E−02
	LOC105679194	Muscle segmentation homeobox‐like (LOC105679194)	−1.98	3.61E−05	1.05E−02
	LOC105679722	Uncharacterized LOC105679722 (LOC105679722)	−2.06	2.27E−04	3.92E−02
	LOC105670118	Uncharacterized methyltransferase‐like protein SPBC21C3.07c (LOC105670118)	−2.07	5.80E−04	8.72E−02
	LOC105667917	Protein crumbs‐like (LOC105667917)	−2.45	1.06E−04	2.33E−02
	LOC105670857	Leucine‐rich repeat‐containing protein egg‐6 (LOC105670857)	−2.49	6.19E−05	1.59E−02
	LOC105671854	Uncharacterized LOC105671854 (LOC105671854)	−3.69	3.19E−09	3.22E−06
	LOC105672186	Uncharacterized LOC105672186 (LOC105672186)	−6.09	3.40E−04	5.78E−02
	LOC105668152	Uncharacterized LOC105668152 (LOC105668152)	−6.68	1.66E−12	2.19E−09
	LOC105675449	Uncharacterized LOC105675449 (LOC105675449)	−8.83	2.13E−13	3.74E−10
	LOC105670525	Chondroadherin‐like (LOC105670525)	−9.31	8.47E−30	8.92E−26
	LOC105668043	Uncharacterized LOC105668043 (LOC105668043)	−10.32	3.15E−18	1.66E−14

**Table A2:** Results of gene ontology enrichment analysis

GO_name	GO_Category	FDR	*p*‐Value
*Enrichment of up‐regulated genes in bacteria‐injected queens in social environment at 4 hpi*			
Serine‐type endopeptidase inhibitor activity	MOLECULAR_FUNCTION	1.13E−33	2.00E−37
Endopeptidase inhibitor activity	MOLECULAR_FUNCTION	6.65E−33	3.51E−36
Endopeptidase regulator activity	MOLECULAR_FUNCTION	6.65E−33	3.51E−36
Peptidase inhibitor activity	MOLECULAR_FUNCTION	8.42E−32	7.41E−35
Peptidase regulator activity	MOLECULAR_FUNCTION	8.42E−32	7.41E−35
Enzyme inhibitor activity	MOLECULAR_FUNCTION	8.24E−30	8.70E−33
Enzyme regulator activity	MOLECULAR_FUNCTION	1.89E−20	2.33E−23
Molecular function regulator	MOLECULAR_FUNCTION	4.10E−17	5.77E−20
Metallopeptidase activity	MOLECULAR_FUNCTION	1.07E−10	1.69E−13
Peptidase activity, acting on l‐amino acid peptides	MOLECULAR_FUNCTION	1.80E−06	3.16E−09
Peptidase activity	MOLECULAR_FUNCTION	3.77E−06	7.30E−09
Proteolysis	BIOLOGICAL_PROCESS	1.21E−04	2.54E−07
Metalloendopeptidase activity	MOLECULAR_FUNCTION	0.0088	2.01E−05
Transition metal ion binding	MOLECULAR_FUNCTION	0.0466	1.15E−04
*Enrichment of down‐regulated genes in bacteria‐injected isolated queens at 2 hpi*			
Serine‐type endopeptidase inhibitor activity	MOLECULAR_FUNCTION	9.33E−24	1.64E−27
Endopeptidase inhibitor activity	MOLECULAR_FUNCTION	2.11E−23	1.12E−26
Endopeptidase regulator activity	MOLECULAR_FUNCTION	2.11E−23	1.12E−26
Peptidase inhibitor activity	MOLECULAR_FUNCTION	9.97E−23	8.77E−26
Peptidase regulator activity	MOLECULAR_FUNCTION	9.97E−23	8.77E−26
Enzyme inhibitor activity	MOLECULAR_FUNCTION	2.17E−21	2.29E−24
Enzyme regulator activity	MOLECULAR_FUNCTION	8.09E−15	9.96E−18
Metallopeptidase activity	MOLECULAR_FUNCTION	5.74E−13	8.08E−16
Molecular function regulator	MOLECULAR_FUNCTION	1.73E−12	2.74E−15
Peptidase activity, acting on l‐amino acid peptides	MOLECULAR_FUNCTION	2.26E−07	3.98E−10
Peptidase activity	MOLECULAR_FUNCTION	4.47E−07	8.65E−10
Proteolysis	BIOLOGICAL_PROCESS	2.95E−06	6.23E−09
Metalloendopeptidase activity	MOLECULAR_FUNCTION	3.96E−05	9.05E−08
Zinc ion binding	MOLECULAR_FUNCTION	5.47E−04	1.35E−06
Hydrolase activity	MOLECULAR_FUNCTION	0.0016	4.33E−06
Transition metal ion binding	MOLECULAR_FUNCTION	0.0032	8.95E−06
Molybdopterin cofactor metabolic process	BIOLOGICAL_PROCESS	0.0403	1.35E−04
Prosthetic group metabolic process	BIOLOGICAL_PROCESS	0.0403	1.35E−04
Molybdopterin cofactor biosynthetic process	BIOLOGICAL_PROCESS	0.0403	1.35E−04
Endopeptidase activity	MOLECULAR_FUNCTION	0.0479	1.77E−04
Exopeptidase activity	MOLECULAR_FUNCTION	0.0479	1.77E−04
*Enrichment of down‐regulated genes in bacteria‐injected isolated queens at 12 hpi*			
Oxidoreductase activity	MOLECULAR_FUNCTION	0.0216	7.60E−06
Oxidation‐reduction process	BIOLOGICAL_PROCESS	0.0216	7.49E−06
*Enrichment of up‐regulated genes in Ringer‐injected isolated queens at 12 hpi*			
Single‐organism metabolic process	BIOLOGICAL_PROCESS	0.0024	4.28E−07
Carbohydrate kinase activity	MOLECULAR_FUNCTION	0.0120	6.36E−06
Carbohydrate phosphorylation	BIOLOGICAL_PROCESS	0.0120	4.47E−06
Cellular glucose homeostasis	BIOLOGICAL_PROCESS	0.0310	4.37E−05
Glucose binding	MOLECULAR_FUNCTION	0.0310	4.37E−05
Carbohydrate homeostasis	BIOLOGICAL_PROCESS	0.0310	4.37E−05
Hexokinase activity	MOLECULAR_FUNCTION	0.0310	4.37E−05
Glucose homeostasis	BIOLOGICAL_PROCESS	0.0310	4.37E−05

**Table A3:** Differentially expressed genes in bacteria‐injected social queens with false discovery rate <10%. T2, T4, T8, and T12 indicate the post‐injection time points

Time point	Locus	Description	LFC	*p*‐Value	Adjusted *p*‐value
T2	LOC105671203	Uncharacterized LOC105671203 (LOC105671203)	3.69	7.03E−13	3.11E−09
	LOC105668751	Uncharacterized LOC105668751 (LOC105668751) (Naickin‐2)	3.06	3.29E−08	7.27E−05
	LOC105670685	Glycine‐rich RNA‐binding protein 1‐like (LOC105670685)	2.45	1.41E−04	9.84E−02
	LOC105677480	Uncharacterized LOC105677480 (LOC105677480)	2.29	1.74E−06	2.56E−03
	LOC105677479	Uncharacterized LOC105677479 (LOC105677479)	2.24	1.45E−04	9.84E−02
	LOC105675449	Uncharacterized LOC105675449 (LOC105675449)	1.79	3.19E−05	3.52E−02
	LOC105676587	Trypsin‐like (LOC105676587)	1.73	9.33E−05	9.17E−02
	LOC105674885	Purine nucleoside phosphorylase‐like (LOC105674885)	−1.99	1.41E−04	9.84E−02
	LOC105678691	Scavenger receptor class B member 1‐like (LOC105678691)	−2.28	1.65E−05	2.08E−02
	LOC105668207	Chymotrypsin‐1‐like (LOC105668207)	−4.01	9.23E−14	8.16E−10
	LOC105677784	Uncharacterized LOC105677784 (LOC105677784)	−4.12	5.07E−10	1.49E−06
	LOC105668184	Uncharacterized LOC105668184 (LOC105668184)	−22.70	1.35E−04	9.84E−02
	LOC105670097	Spidroin‐1 (LOC105670097)	−26.14	7.82E−07	1.38E−03
T4	LOC105670077	DNA repair protein RAD50‐like (LOC105670077)	9.96	2.39E−06	4.18E−04
	LOC105670097	Spidroin‐1 (LOC105670097)	9.00	6.39E−39	3.58E−35
	LOC105679457	Uncharacterized LOC105679457 (LOC105679457)	8.37	1.61E−04	1.54E−02
	LOC105679565	A disintegrin and metalloproteinase with thrombospondin motifs 4‐like (LOC105679565)	7.51	1.67E−03	9.52E−02
	LOC105679203	Cytochrome P450 4g15‐like (LOC105679203)	7.21	6.19E−11	4.34E−08
	LOC105671203	Uncharacterized LOC105671203 (LOC105671203)	6.80	1.90E−72	2.13E−68
	LOC105677347	Uncharacterized LOC105677347 (LOC105677347)	6.21	2.74E−05	3.41E−03
	LOC105668553	A disintegrin and metalloproteinase with thrombospondin motifs 2‐like (LOC105668553)	5.68	7.90E−04	5.11E−02
	LOC105677333	Uncharacterized LOC105677333 (LOC105677333)	5.43	5.89E−07	1.32E−04
	LOC105676228	Glutamyl aminopeptidase‐like (LOC105676228)	5.37	3.28E−05	3.89E−03
	LOC105677348	Uncharacterized LOC105677348 (LOC105677348)	5.28	1.98E−18	3.70E−15
	LOC105677332	Uncharacterized LOC105677332 (LOC105677332)	5.23	7.15E−12	6.16E−09
	LOC105671207	A disintegrin and metalloproteinase with thrombospondin motifs 7‐like (LOC105671207)	5.20	6.21E−05	6.82E−03
	LOC105670873	Uncharacterized LOC105670873 (LOC105670873)	4.97	2.43E−04	2.01E−02
	LOC105674384	Uncharacterized LOC105674384 (LOC105674384)	4.95	2.04E−04	1.82E−02
	LOC105676577	Glutamyl aminopeptidase‐like (LOC105676577)	4.81	2.18E−04	1.88E−02
	LOC105674526	Uncharacterized LOC105674526 (LOC105674526)	4.80	5.82E−07	1.32E−04
	LOC105676006	Uncharacterized LOC105676006 (LOC105676006)	4.62	9.75E−10	5.20E−07
	LOC105678717	Uncharacterized LOC105678717 (LOC105678717)	4.60	1.09E−03	6.68E−02
	LOC105676008	Uncharacterized LOC105676008 (LOC105676008)	4.47	2.62E−05	3.34E−03
	LOC105670188	Luciferin 4‐monooxygenase‐like (LOC105670188)	4.37	3.59E−09	1.68E−06
	LOC105671772	Uncharacterized LOC105671772 (LOC105671772)	4.28	5.57E−04	3.81E−02
	LOC105674010	Phosphotriesterase‐related protein‐like (LOC105674010)	4.14	1.41E−04	1.37E−02
	LOC105673254	Thyrotropin‐releasing hormone‐degrading ectoenzyme‐like (LOC105673254)	4.11	2.68E−04	2.19E−02
	LOC105672921	Zinc finger protein 468‐like (LOC105672921)	4.10	1.10E−03	6.71E−02
	LOC105672283	Uncharacterized threonine‐rich GPI‐anchored glycoprotein PJ4664.02‐like (LOC105672283)	4.04	6.91E−06	1.08E−03
	LOC105679148	Uncharacterized LOC105679148 (LOC105679148)	3.98	6.36E−07	1.40E−04
	LOC105678936	Uncharacterized LOC105678936 (LOC105678936)	3.92	1.05E−09	5.37E−07
	LOC105671306	A disintegrin and metalloproteinase with thrombospondin motifs 17‐like (LOC105671306)	3.83	4.76E−04	3.37E−02
	LOC105674631	Uncharacterized LOC105674631 (LOC105674631)	3.82	3.84E−04	2.89E−02
	LOC105679175	Uncharacterized LOC105679175 (LOC105679175)	3.73	4.82E−06	7.60E−04
	LOC105671771	Uncharacterized LOC105671771 (LOC105671771)	3.57	5.05E−05	5.66E−03
	LOC105679161	A disintegrin and metalloproteinase with thrombospondin motifs 17‐like (LOC105679161)	3.52	7.78E−04	5.08E−02
	LOC105674893	Uncharacterized LOC105674893 (LOC105674893)	3.48	5.07E−04	3.55E−02
	LOC105670869	Uncharacterized LOC105670869 (LOC105670869)	3.29	1.47E−10	9.16E−08
	LOC105670598	A disintegrin and metalloproteinase with thrombospondin motifs 12‐like (LOC105670598)	3.28	2.82E−04	2.26E−02
	LOC105673458	Thyrotropin‐releasing hormone‐degrading ectoenzyme‐like (LOC105673458)	3.18	2.90E−06	4.84E−04
	LOC105668847	Uncharacterized LOC105668847 (LOC105668847)	3.14	1.58E−03	9.20E−02
	LOC105674525	Uncharacterized LOC105674525 (LOC105674525)	3.12	3.41E−06	5.62E−04
	LOC105679050	Transcription termination factor 2‐like (LOC105679050)	3.10	9.41E−09	3.63E−06
	LOC105668490	Uncharacterized LOC105668490 (LOC105668490)	2.85	9.94E−05	1.03E−02
	LOC105670243	Uncharacterized LOC105670243 (LOC105670243)	2.81	2.94E−07	7.38E−05
	LOC105678344	Gephyrin‐like (LOC105678344)	2.77	3.07E−05	3.70E−03
	LOC105674779	Fatty acid synthase‐like (LOC105674779)	2.72	1.61E−14	2.00E−11
	LOC105671607	Uncharacterized LOC105671607 (LOC105671607)	2.69	1.24E−03	7.41E−02
	LOC105675620	Aminopeptidase N‐like (LOC105675620)	2.64	1.15E−07	3.22E−05
	LOC105672800	Uncharacterized LOC105672800 (LOC105672800)	2.64	3.30E−05	3.89E−03
	LOC105678986	Uncharacterized LOC105678986 (LOC105678986)	2.64	5.65E−08	1.71E−05
	LOC105674397	Uncharacterized LOC105674397 (LOC105674397)	2.60	4.40E−08	1.37E−05
	LOC105670868	Uncharacterized LOC105670868 (LOC105670868)	2.56	2.31E−08	8.08E−06
	LOC105669605	A disintegrin and metalloproteinase with thrombospondin motifs 20‐like (LOC105669605)	2.51	1.21E−03	7.34E−02
	LOC105670022	Chymotrypsin‐2‐like (LOC105670022)	2.47	3.77E−12	3.84E−09
	LOC105679174	Uncharacterized LOC105679174 (LOC105679174)	2.41	2.83E−04	2.26E−02
	LOC105670219	Uncharacterized LOC105670219 (LOC105670219)	2.38	4.34E−04	3.10E−02
	LOC105674484	RCC1 and BTB domain‐containing protein 1‐like (LOC105674484)	2.35	3.58E−04	2.78E−02
	LOC105670871	Uncharacterized LOC105670871 (LOC105670871)	2.34	3.47E−05	4.04E−03
	LOC105670872	Uncharacterized LOC105670872 (LOC105670872)	2.31	1.42E−06	2.65E−04
	LOC105668795	Flavin‐containing monooxygenase FMO GS‐OX‐like 3 (LOC105668795)	2.30	7.32E−09	3.04E−06
	LOC105667907	Thyroid receptor‐interacting protein 11‐like (LOC105667907)	2.29	6.90E−07	1.43E−04
	LOC105668248	Uncharacterized LOC105668248 (LOC105668248)	2.28	1.80E−05	2.41E−03
	LOC105670430	Aminopeptidase N‐like (LOC105670430)	2.27	2.06E−05	2.68E−03
	LOC105673464	Venom carboxylesterase‐6‐like (pseudo)	2.27	4.42E−08	1.37E−05
	LOC105678987	Uncharacterized LOC105678987 (LOC105678987)	2.24	2.97E−07	7.38E−05
	LOC105670899	Uncharacterized LOC105670899 (LOC105670899)	2.23	6.89E−07	1.43E−04
	LOC105679177	Uncharacterized LOC105679177 (LOC105679177)	2.21	2.08E−04	1.83E−02
	LOC105674677	Uncharacterized LOC105674677 (LOC105674677)	2.20	1.81E−07	4.83E−05
	LOC105674740	Zinc carboxypeptidase‐like (LOC105674740)	2.16	7.34E−06	1.11E−03
	LOC105674403	Uncharacterized LOC105674403 (LOC105674403)	2.13	1.16E−03	7.09E−02
	LOC105671625	Uncharacterized LOC105671625 (LOC105671625)	2.10	1.12E−04	1.12E−02
	LOC105678984	Uncharacterized LOC105678984 (LOC105678984)	2.08	8.18E−07	1.64E−04
	LOC105676005	Uncharacterized LOC105676005 (LOC105676005)	1.99	2.92E−04	2.31E−02
	LOC105668487	Uncharacterized LOC105668487 (LOC105668487)	1.97	2.73E−05	3.41E−03
	LOC105674716	Zinc carboxypeptidase A 1‐like (LOC105674716)	1.92	1.45E−05	2.03E−03
	LOC105672402	Fatty acid synthase‐like (LOC105672402)	1.91	7.20E−06	1.11E−03
	LOC105674892	Uncharacterized LOC105674892 (LOC105674892)	1.91	3.83E−04	2.89E−02
	LOC105670241	Uncharacterized LOC105670241 (LOC105670241)	1.90	7.66E−06	1.13E−03
	LOC105676597	Uncharacterized LOC105676597 (LOC105676597)	1.87	1.43E−08	5.17E−06
	LOC105678934	Uncharacterized LOC105678934 (LOC105678934)	1.86	2.95E−05	3.59E−03
	LOC105671177	l‐xylulose reductase‐like (LOC105671177)	1.85	1.63E−04	1.55E−02
	LOC105671446	A disintegrin and metalloproteinase with thrombospondin motifs 7‐like (LOC105671446)	1.85	5.42E−04	3.75E−02
	LOC105670897	Uncharacterized LOC105670897 (LOC105670897)	1.85	9.23E−08	2.72E−05
	LOC105674728	Muscle M‐line assembly protein unc‐89‐like (LOC105674728)	1.84	4.24E−04	3.08E−02
	LOC105678217	Uncharacterized LOC105678217 (LOC105678217)	1.84	1.42E−07	3.88E−05
	LOC105668988	Uncharacterized LOC105668988 (LOC105668988)	1.83	5.66E−10	3.17E−07
	LOC105679014	Uncharacterized LOC105679014 (LOC105679014)	1.83	1.73E−04	1.60E−02
	LOC105671241	Aminopeptidase N‐like (LOC105671241)	1.83	4.86E−05	5.50E−03
	LOC105670900	Uncharacterized LOC105670900 (LOC105670900)	1.82	1.23E−04	1.22E−02
	LOC105678353	Aminopeptidase N‐like (LOC105678353)	1.81	8.06E−04	5.19E−02
	LOC105676303	N‐(5‐amino‐5‐carboxypentanoyl)‐l‐cysteinyl‐d‐valine synthase (LOC105676303)	1.81	6.33E−09	2.73E−06
	LOC105670239	Uncharacterized LOC105670239 (LOC105670239)	1.79	2.90E−06	4.84E−04
	LOC105674406	Uncharacterized LOC105674406 (LOC105674406)	1.78	2.50E−04	2.06E−02
	LOC105678342	Gephyrin‐like (LOC105678342)	1.77	1.53E−05	2.12E−03
	LOC105679156	Uncharacterized LOC105679156 (LOC105679156)	1.76	1.78E−04	1.63E−02
	LOC105668244	Uncharacterized LOC105668244 (LOC105668244)	1.75	9.96E−07	1.92E−04
	LOC105672123	Uncharacterized LOC105672123 (LOC105672123)	1.74	1.01E−03	6.24E−02
	LOC105667677	Zinc carboxypeptidase‐like (LOC105667677)	1.72	2.13E−10	1.26E−07
	LOC105676007	Uncharacterized LOC105676007 (LOC105676007)	1.70	5.98E−04	4.06E−02
	LOC105678817	Protein toll‐like (LOC105678817)	1.69	1.02E−04	1.05E−02
	LOC105670238	Uncharacterized LOC105670238 (LOC105670238)	1.67	7.51E−06	1.12E−03
	LOC105677480	Uncharacterized LOC105677480 (LOC105677480)	1.66	9.46E−07	1.86E−04
	LOC105677501	Venom acid phosphatase Acph‐1‐like (LOC105677501)	1.66	1.03E−04	1.05E−02
	LOC105670214	Tissue factor pathway inhibitor‐like (LOC105670214)	1.60	8.25E−05	8.80E−03
	LOC105673951	Chymotrypsin‐1‐like (LOC105673951)	1.59	6.92E−07	1.43E−04
	LOC105668779	Troponin C	1.55	8.95E−06	1.30E−03
	LOC105675453	Serine racemase‐like (LOC105675453)	1.55	6.50E−04	4.39E−02
	LOC105676003	Uncharacterized LOC105676003 (LOC105676003)	1.53	1.96E−04	1.78E−02
	LOC105676004	Uncharacterized LOC105676004 (LOC105676004)	1.52	8.36E−04	5.32E−02
	LOC105676346	Uncharacterized LOC105676346 (LOC105676346)	1.52	2.24E−06	3.99E−04
	LOC105678648	Protein toll‐like (LOC105678648)	1.50	5.55E−04	3.81E−02
	LOC105667368	46 kDa FK506‐binding nuclear protein‐like (LOC105667368)	1.49	4.87E−04	3.43E−02
	LOC105667585	Laccase‐1‐like (LOC105667585)	1.48	9.34E−05	9.87E−03
	LOC105670240	Uncharacterized LOC105670240 (LOC105670240)	1.47	1.27E−04	1.25E−02
	LOC105668195	Uncharacterized LOC105668195 (LOC105668195)	1.45	3.80E−07	8.88E−05
	LOC105671544	Isthmin‐like (LOC105671544)	1.44	2.33E−04	1.95E−02
	LOC105679722	Uncharacterized LOC105679722 (LOC105679722)	1.43	2.16E−04	1.88E−02
	LOC105670898	Uncharacterized LOC105670898 (LOC105670898)	1.43	2.15E−04	1.88E−02
	LOC105674374	Uncharacterized LOC105674374 (LOC105674374)	1.42	4.32E−04	3.10E−02
	LOC105675133	Mitochondrial uncoupling protein 2‐like (LOC105675133)	1.42	2.67E−07	6.96E−05
	LOC105672982	Chromosome‐associated kinesin KIF4 (LOC105672982)	1.39	7.11E−05	7.73E−03
	LOC105677706	Bipolar kinesin KRP‐130‐like (LOC105677706)	1.37	6.99E−04	4.69E−02
	LOC105677471	Facilitated trehalose transporter Tret1‐like (LOC105677471)	1.33	3.92E−05	4.53E−03
	LOC105670134	Uncharacterized LOC105670134 (LOC105670134)	1.32	1.59E−03	9.20E−02
	LOC105677684	E3 ubiquitin‐protein ligase TRIM71 (LOC105677684)	1.28	1.59E−05	2.17E−03
	LOC105672089	Lipid storage droplets surface‐binding protein 2‐like (LOC105672089)	1.28	2.24E−06	3.99E−04
	LOC105671386	Organic cation transporter 1‐like (LOC105671386)	1.25	9.68E−04	6.03E−02
	LOC105675373	Cyclin‐dependent kinase 4 (LOC105675373)	1.24	2.52E−05	3.25E−03
	LOC105669176	Organic cation transporter protein‐like (LOC105669176)	1.22	1.69E−04	1.59E−02
	LOC105671746	Probable cytochrome P450 304a1 (LOC105671746)	1.22	2.24E−04	1.90E−02
	LOC105671768	Uncharacterized LOC105671768 (LOC105671768)	1.15	5.39E−04	3.75E−02
	LOC105668043	Uncharacterized LOC105668043 (LOC105668043)	1.13	1.31E−03	7.71E−02
	LOC105668144	1‐acyl‐sn‐glycerol‐3‐phosphate acyltransferase alpha‐like (LOC105668144)	1.09	7.80E−04	5.08E−02
	LOC105675016	Peroxidase‐like (LOC105675016)	1.01	3.90E−04	2.91E−02
	LOC105677622	Uncharacterized LOC105677622 (LOC105677622)	0.99	1.63E−03	9.34E−02
	LOC105676083	Baculoviral IAP repeat‐containing protein 7‐A‐like (LOC105676083)	0.97	7.21E−04	4.81E−02
	LOC105668364	Mesencephalic astrocyte‐derived neurotrophic factor homolog (LOC105668364)	0.91	1.74E−03	9.83E−02
	LOC105668947	Endoplasmin (LOC105668947)	0.83	1.69E−03	9.60E−02
	LOC105669096	Spondin‐1 (LOC105669096)	−0.90	1.22E−03	7.34E−02
	LOC105668946	Uncharacterized LOC105668946 (LOC105668946)	−0.96	7.27E−04	4.82E−02
	LOC105673575	Venom metalloproteinase 3‐like (LOC105673575)	−0.98	3.16E−04	2.47E−02
	LOC105670254	Glycine‐rich cell wall structural protein‐like (LOC105670254)	−1.00	2.02E−04	1.81E−02
	LOC105672527	Venom allergen 3‐like (LOC105672527)	−1.00	2.70E−04	2.19E−02
	LOC105670714	Alpha‐amylase A (LOC105670714)	−1.01	3.79E−04	2.88E−02
	LOC105672704	Receptor‐type tyrosine‐protein phosphatase epsilon‐like (LOC105672704)	−1.04	8.56E−04	5.42E−02
	LOC105675546	Uncharacterized LOC105675546 (LOC105675546)	−1.05	8.23E−04	5.27E−02
	LOC105676883	Serine hydroxymethyltransferase	−1.08	4.27E−04	3.08E−02
	LOC105676184	Troponin C	−1.09	2.32E−04	1.95E−02
	LOC105678432	Acetyl‐CoA carboxylase (LOC105678432)	−1.11	9.56E−04	6.02E−02
	LOC105671942	Hexokinase‐2‐like (LOC105671942)	−1.12	9.69E−04	6.03E−02
	LOC105672608	Facilitated trehalose transporter Tret1‐like (LOC105672608)	−1.14	7.47E−04	4.92E−02
	LOC105678761	Serine protease inhibitor 3/4‐like (LOC105678761)	−1.17	4.04E−04	2.98E−02
	LOC105675773	Peptidoglycan‐recognition protein SC2‐like (LOC105675773)	−1.20	9.78E−06	1.40E−03
	LOC105669805	Facilitated trehalose transporter Tret1‐2 homolog (LOC105669805)	−1.22	4.34E−05	4.97E−03
	LOC105674040	Apolipophorins (LOC105674040)	−1.23	4.34E−06	7.05E−04
	LOC105679189	Uncharacterized LOC105679189 (LOC105679189)	−1.28	4.03E−04	2.98E−02
	LOC105677003	Aminopeptidase N‐like (LOC105677003)	−1.28	1.09E−04	1.10E−02
	LOC105668704	Organic cation transporter protein (LOC105668704)	−1.30	1.95E−05	2.57E−03
	LOC105670974	Uncharacterized LOC105670974 (LOC105670974)	−1.30	1.87E−06	3.43E−04
	LOC105674681	Cytochrome P450 6j1‐like (LOC105674681)	−1.31	1.57E−04	1.51E−02
	LOC105677248	Protein phosphatase 1 regulatory subunit 3C‐B (LOC105677248)	−1.34	7.82E−05	8.42E−03
	LOC105672092	Uncharacterized LOC105672092 (LOC105672092)	−1.35	9.67E−05	1.01E−02
	LOC105675916	Major royal jelly protein 5‐like (LOC105675916)	−1.37	1.39E−06	2.63E−04
	LOC105672956	Ribose‐phosphate pyrophosphokinase 1 (LOC105672956)	−1.39	3.22E−07	7.68E−05
	LOC105676485	Uncharacterized LOC105676485 (LOC105676485)	−1.40	2.24E−04	1.90E−02
	LOC105672335	Uncharacterized LOC105672335 (LOC105672335)	−1.41	3.70E−04	2.85E−02
	LOC105667549	Vascular endothelial growth factor receptor 1‐like (LOC105667549)	−1.42	5.42E−05	6.01E−03
	LOC105676142	Vascular endothelial growth factor receptor kdr‐like (LOC105676142)	−1.45	1.59E−03	9.20E−02
	LOC105669099	Vascular endothelial growth factor B‐like (LOC105669099)	−1.45	4.09E−04	2.99E−02
	LOC105674346	Neural‐cadherin (LOC105674346)	−1.49	3.92E−08	1.29E−05
	LOC105671703	Facilitated trehalose transporter Tret1‐like (LOC105671703)	−1.52	1.39E−03	8.15E−02
	LOC105672628	Esterase FE4‐like (LOC105672628)	−1.53	5.18E−09	2.32E−06
	LOC105670266	Sodium/potassium/calcium exchanger 3‐like (LOC105670266)	−1.54	1.72E−04	1.60E−02
	LOC105679180	Solute carrier family 2	−1.59	3.18E−07	7.68E−05
	LOC105671469	d‐arabinitol dehydrogenase 1‐like (LOC105671469)	−1.66	1.69E−09	8.22E−07
	LOC105675436	Chymotrypsin inhibitor‐like (LOC105675436)	−1.69	1.42E−10	9.16E−08
	LOC105669509	Arylsulfatase B‐like (LOC105669509)	−1.71	4.52E−06	7.23E−04
	LOC105669158	Sorbitol dehydrogenase‐like (LOC105669158)	−1.71	8.01E−09	3.21E−06
	LOC105675717	Defensing‐2 (LOC105675717)	−1.80	1.05E−08	3.93E−06
	LOC105679484	Sorbitol dehydrogenase‐like (LOC105679484)	−1.80	3.71E−04	2.85E−02
	LOC105679620	Uncharacterized LOC105679620 (LOC105679620)	−1.88	1.68E−05	2.26E−03
	LOC105679945	Cytochrome P450 4C1‐like (LOC105679945)	−1.95	1.30E−03	7.71E−02
	LOC105679847	Probable phytanoyl‐CoA dioxygenase (LOC105679847)	−2.03	2.39E−11	1.92E−08
	LOC105667920	Phosphoenolpyruvate carboxykinase [GTP]‐like (LOC105667920)	−2.08	1.30E−05	1.84E−03
	LOC105675714	Uncharacterized LOC105675714 (LOC105675714)	−2.10	2.85E−04	2.27E−02
	LOC105672537	Alpha‐glucosidase‐like (LOC105672537)	−2.15	4.98E−15	6.98E−12
	LOC105672219	Leucine‐rich repeat‐containing protein 4‐like (LOC105672219)	−2.17	3.72E−11	2.78E−08
	LOC105680018	Uncharacterized LOC105680018 (LOC105680018)	−2.20	2.77E−06	4.78E−04
	LOC105675643	Prismalin‐14‐like (LOC105675643)	−2.29	2.95E−05	3.59E−03
	LOC105678258	Facilitated trehalose transporter Tret1‐like (LOC105678258)	−2.35	1.24E−03	7.41E−02
	LOC105668871	Phenoloxidase 2‐like (LOC105668871)	−2.36	8.57E−17	1.37E−13
	LOC105676062	Protein henna (LOC105676062)	−2.37	3.44E−08	1.17E−05
	LOC105679763	Uncharacterized oxidoreductase C26H5.09c‐like (LOC105679763)	−2.40	4.11E−13	4.60E−10
	LOC105671889	Synaptobrevin homolog YKT6‐like (LOC105671889)	−2.44	7.03E−07	1.43E−04
	LOC105667386	Hexamerin‐like (LOC105667386)	−2.63	4.83E−12	4.51E−09
	LOC105675433	Dynein beta chain, ciliary (LOC105675433)	−2.97	1.07E−07	3.08E−05
	LOC105674885	Purine nucleoside phosphorylase‐like (LOC105674885)	−3.03	5.32E−23	1.19E−19
	LOC105674055	Uncharacterized protein DDB_G0290685‐like (LOC105674055)	−3.04	1.43E−27	4.00E−24
	LOC105674482	Aldehyde dehydrogenase family 1 member A3 (LOC105674482)	−3.25	9.19E−34	3.43E−30
	LOC105676600	Arylphorin subunit alpha‐like (LOC105676600)	−3.51	1.97E−04	1.78E−02
T8	LOC105668186	Uncharacterized LOC105668186 (LOC105668186)	8.25	4.34E−04	8.24E−02
	LOC105670097	Spidroin‐1 (LOC105670097)	6.79	5.27E−07	3.32E−04
	LOC105672689	Uncharacterized LOC105672689 (LOC105672689)	2.28	3.82E−09	3.81E−06
	LOC105675162	Uncharacterized LOC105675162 (LOC105675162)	1.97	1.12E−11	1.67E−08
	LOC105670021	Chymotrypsin‐2‐like (LOC105670021)	1.78	8.18E−05	2.33E−02
	LOC105667585	Laccase‐1‐like (LOC105667585)	1.66	2.68E−12	5.00E−09
	LOC105671850	Protein‐S‐isoprenylcysteine O‐methyltransferase (LOC105671850)	1.56	2.02E−05	7.47E−03
	LOC105669469	Cytochrome b5‐like (LOC105669469)	1.44	5.07E−06	2.33E−03
	LOC105671337	Putative fatty acyl‐CoA reductase CG5065 (LOC105671337)	1.39	2.23E−06	1.21E−03
	LOC105672439	Alpha‐sarcoglycan (LOC105672439)	1.37	3.77E−05	1.29E−02
	LOC105671866	Serine protease gd‐like (LOC105671866)	1.29	2.23E−07	1.48E−04
	LOC105673874	2‐oxoisovalerate dehydrogenase subunit beta	1.26	3.26E−04	6.90E−02
	LOC105673821	Vascular endothelial growth factor A‐like (LOC105673821)	1.26	4.54E−05	1.43E−02
	LOC105669027	Putative nuclease HARBI1 (pseudo)	1.19	3.03E−04	6.71E−02
	LOC105673881	Beta‐1,3‐glucan‐binding protein‐like (LOC105673881)	1.18	1.43E−05	5.70E−03
	LOC105674055	Uncharacterized protein DDB_G0290685‐like (LOC105674055)	1.10	2.83E−10	3.76E−07
	LOC105677650	Speckle targeted PIP5K1A‐regulated poly(A) polymerase‐like (LOC105677650)	1.10	2.68E−04	6.04E−02
	LOC105675725	Serine protease snake‐like (LOC105675725)	1.04	1.68E−04	4.19E−02
	LOC105667656	Uncharacterized LOC105667656 (LOC105667656)	1.01	2.12E−05	7.47E−03
	LOC105676251	Uncharacterized LOC105676251 (LOC105676251)	0.99	2.00E−04	4.89E−02
	LOC105680007	Protein toll (LOC105680007)	0.98	3.45E−04	7.12E−02
	LOC105672359	Thiamine transporter 2‐like (LOC105672359)	0.97	3.29E−04	6.90E−02
	LOC105673986	G‐protein coupled receptor moody (LOC105673986)	0.96	5.07E−04	9.06E−02
	LOC105669664	Uncharacterized LOC105669664 (LOC105669664)	0.92	3.78E−04	7.67E−02
	LOC105674673	Putative histone‐lysine N‐methyltransferase 1 (LOC105674673)	0.89	5.02E−04	9.06E−02
	LOC105676303	N‐(5‐amino‐5‐carboxypentanoyl)‐l‐cysteinyl‐d‐valine synthase (LOC105676303)	0.86	2.17E−04	5.18E−02
	LOC105673707	Kinesin‐like protein unc‐104 (LOC105673707)	0.84	1.10E−04	2.99E−02
	LOC105669176	Organic cation transporter protein‐like (LOC105669176)	0.84	5.60E−06	2.48E−03
	LOC105672117	ATP‐binding cassette sub‐family G member 4 (LOC105672117)	0.83	1.35E−05	5.56E−03
	LOC105678395	Uncharacterized LOC105678395 (LOC105678395)	0.82	4.07E−04	8.09E−02
	LOC105671203	Uncharacterized LOC105671203 (LOC105671203)	0.76	1.31E−04	3.40E−02
	LOC105671406	Putative phosphatidate phosphatase (LOC105671406)	0.72	4.17E−04	8.09E−02
	LOC105676136	Guanine nucleotide‐binding protein G(i) subunit alpha (LOC105676136)	0.69	1.99E−05	7.47E−03
	LOC105671687	Plastin‐2 (LOC105671687)	0.66	5.64E−04	9.93E−02
	LOC105673509	Ankyrin repeat and BTB/POZ domain‐containing protein BTBD11 (LOC105673509)	0.58	2.40E−04	5.63E−02
	LOC105679739	Uncharacterized LOC105679739 (LOC105679739)	−0.59	2.11E−05	7.47E−03
	LOC105678332	Pancreatic triacylglycerol lipase‐like (LOC105678332)	−0.63	7.11E−05	2.16E−02
	LOC105677611	Uncharacterized LOC105677611 (LOC105677611)	−0.68	4.30E−05	1.39E−02
	LOC105671942	Hexokinase‐2‐like (LOC105671942)	−0.71	4.58E−04	8.56E−02
	LOC105669509	Arylsulfatase B‐like (LOC105669509)	−0.74	2.60E−04	5.98E−02
	LOC105667685	Solute carrier organic anion transporter family member 2A1 (LOC105667685)	−0.74	3.18E−04	6.90E−02
	LOC105678768	Lipoyltransferase 1	−0.81	1.31E−04	3.40E−02
	LOC105674040	Apolipophorins (LOC105674040)	−0.85	7.22E−05	2.16E−02
	LOC105676627	Uncharacterized LOC105676627 (LOC105676627)	−0.97	4.82E−04	8.87E−02
	LOC105667549	Vascular endothelial growth factor receptor 1‐like (LOC105667549)	−1.00	4.19E−04	8.09E−02
	LOC105674437	Aquaporin‐like (LOC105674437)	−1.01	8.98E−07	5.37E−04
	LOC105672704	Receptor‐type tyrosine‐protein phosphatase epsilon‐like (LOC105672704)	−1.03	9.68E−05	2.69E−02
	LOC105675201	Glycine N‐methyltransferase (LOC105675201)	−1.03	2.68E−08	2.29E−05
	LOC105676399	Netrin receptor UNC5C‐like (LOC105676399)	−1.04	2.23E−07	1.48E−04
	LOC105675916	Major royal jelly protein 5‐like (LOC105675916)	−1.08	8.13E−05	2.33E−02
	LOC105672628	Esterase FE4‐like (LOC105672628)	−1.13	4.28E−08	3.42E−05
	LOC105669186	Uncharacterized LOC105669186 (LOC105669186)	−1.14	4.00E−05	1.33E−02
	LOC105676405	Fatty acid synthase (LOC105676405)	−1.18	2.45E−09	2.67E−06
	LOC105678833	Acyl‐CoA Delta(11) desaturase‐like (LOC105678833)	−1.19	2.75E−12	5.00E−09
	LOC105678901	High affinity nerve growth factor receptor‐like (LOC105678901)	−1.25	3.89E−06	1.94E−03
	LOC105675090	Uncharacterized LOC105675090 (LOC105675090)	−1.27	4.99E−06	2.33E−03
	LOC105677615	Alpha‐tocopherol transfer protein‐like (LOC105677615)	−1.42	2.93E−12	5.00E−09
	LOC105671862	Uncharacterized LOC105671862 (LOC105671862)	−1.53	1.52E−04	3.88E−02
	LOC105679194	Muscle segmentation homeobox‐like (LOC105679194)	−1.54	2.47E−06	1.29E−03
	LOC105680137	Tryptophan 2,3‐dioxygenase (LOC105680137)	−1.67	8.66E−13	2.59E−09
	LOC105676142	Vascular endothelial growth factor receptor kdr‐like (LOC105676142)	−1.69	2.22E−07	1.48E−04
	LOC105680118	Cysteine proteinase 1‐like (LOC105680118)	−2.03	7.00E−06	2.99E−03
	LOC105677462	Peroxisomal hydratase‐dehydrogenase‐epimerase‐like (LOC105677462)	−2.07	1.32E−40	1.58E−36
	LOC105680018	Uncharacterized LOC105680018 (LOC105680018)	−2.09	1.08E−09	1.29E−06
	LOC105675840	Uncharacterized protein K02A2.6‐like (LOC105675840)	−3.23	1.08E−13	4.29E−10
	LOC105678364	Uncharacterized LOC105678364 (LOC105678364)	−3.29	1.39E−06	7.92E−04
	LOC105669037	Uncharacterized LOC105669037 (LOC105669037)	−4.30	6.30E−14	3.77E−10
	LOC105670525	Chondroadherin‐like (LOC105670525)	−5.15	1.14E−08	1.05E−05
T12	LOC105669627	Uncharacterized LOC105669627 (LOC105669627)	10.40	9.22E−06	1.27E−02
	LOC105667928	Uncharacterized LOC105667928 (pseudo)	5.40	3.43E−10	2.13E−06
	LOC105669318	Kynurenine/alpha‐aminoadipate aminotransferase	2.96	1.84E−06	2.86E−03
	LOC105673362	Limulus clotting factor C‐like (LOC105673362)	2.74	1.11E−07	3.45E−04
	LOC105676242	Uncharacterized LOC105676242 (LOC105676242)	2.37	1.68E−07	4.16E−04
	LOC105670707	Uncharacterized transmembrane protein DDB_G0289901‐like (LOC105670707)	2.29	2.50E−07	5.16E−04
	LOC105676540	Uncharacterized LOC105676540 (LOC105676540)	2.16	8.06E−07	1.43E−03
	LOC105670591	Uncharacterized LOC105670591 (LOC105670591)	2.09	7.28E−05	6.94E−02
	LOC105677207	Gamma‐glutamyl transpeptidase 1‐like (LOC105677207)	2.04	5.11E−05	5.28E−02
	LOC105677194	Glucose dehydrogenase	1.82	2.74E−05	3.09E−02
	LOC105674352	Aromatic‐l‐amino acid decarboxylase (LOC105674352)	1.78	1.43E−05	1.77E−02
	LOC105677462	Peroxisomal hydratase‐dehydrogenase‐epimerase‐like (LOC105677462)	−3.04	2.10E−14	2.61E−10
	LOC105671854	Uncharacterized LOC105671854 (LOC105671854)	−3.30	5.52E−08	2.28E−04

LFC: log_2_ fold change.

**Table A4:** Differentially expressed genes in bacteria‐injected isolated queens with false discovery rate <10%. T2, T4, T8, and T12 indicate the post‐injection time points

Time point	Locus	Description	LFC	*p*‐Value	Adjusted *p*‐value
T2	LOC105672684	Protein hairy (LOC105672684)	1.75	3.31E−06	1.93E−03
	LOC105672434	Uncharacterized LOC105672434 (LOC105672434)	1.53	2.39E−11	5.13E−08
	LOC105670707	Uncharacterized transmembrane protein DDB_G0289901‐like (LOC105670707)	1.48	2.33E−07	2.27E−04
	LOC105675132	Uncharacterized LOC105675132 (LOC105675132)	1.45	3.35E−04	4.66E−02
	LOC105673362	Limulus clotting factor C‐like (LOC105673362)	1.45	4.68E−07	4.18E−04
	LOC105669805	Facilitated trehalose transporter Tret1‐2 homolog (LOC105669805)	1.20	1.75E−05	6.19E−03
	LOC105679763	Uncharacterized oxidoreductase C26H5.09c‐like (LOC105679763)	1.18	9.17E−04	8.86E−02
	LOC105676470	MAP/microtubule affinity‐regulating kinase 3‐like (LOC105676470)	1.10	9.12E−10	1.40E−06
	LOC105676540	Uncharacterized LOC105676540 (LOC105676540)	1.06	1.79E−05	6.19E−03
	LOC105676242	Uncharacterized LOC105676242 (LOC105676242)	0.96	9.84E−08	1.17E−04
	LOC105668293	Chymotrypsin‐1‐like (LOC105668293)	0.94	7.93E−06	3.81E−03
	LOC105672325	Calcium‐binding mitochondrial carrier protein SCaMC‐2‐like (LOC105672325)	0.90	6.63E−04	7.04E−02
	LOC105671678	Collagen alpha‐2(I) chain‐like (LOC105671678)	0.89	5.94E−05	1.35E−02
	LOC105679973	Fatty acid binding protein 1‐B.1‐like (LOC105679973)	0.82	1.46E−04	2.56E−02
	LOC105677611	Uncharacterized LOC105677611 (LOC105677611)	0.76	1.73E−04	2.86E−02
	LOC105672337	Protein fem‐1 homolog B (LOC105672337)	0.67	4.27E−04	5.51E−02
	LOC105674292	Nose resistant to fluoxetine protein 6‐like (LOC105674292)	0.58	5.93E−04	6.77E−02
	LOC105668729	Nuclear factor NF‐kappa‐B p100 subunit (LOC105668729) (Relish)	0.54	1.01E−03	9.71E−02
	LOC105677684	E3 ubiquitin‐protein ligase TRIM71 (LOC105677684)	−0.72	1.01E−04	2.03E−02
	LOC105678648	Protein toll‐like (LOC105678648)	−0.72	3.01E−04	4.30E−02
	LOC105667719	Uncharacterized protein PFB0145c‐like (LOC105667719)	−0.81	2.73E−04	4.01E−02
	LOC105671848	Putative exonuclease GOR (LOC105671848)	−0.82	1.11E−04	2.12E−02
	LOC105674378	Uncharacterized LOC105674378 (LOC105674378)	−0.82	6.36E−04	6.89E−02
	LOC105676004	Uncharacterized LOC105676004 (LOC105676004)	−0.82	6.60E−04	7.04E−02
	LOC105671329	Insulin‐degrading enzyme‐like (LOC105671329)	−0.83	7.00E−04	7.13E−02
	LOC105672982	Chromosome‐associated kinesin KIF4 (LOC105672982)	−0.84	3.02E−05	8.75E−03
	LOC105670214	Tissue factor pathway inhibitor‐like (LOC105670214)	−0.87	9.09E−04	8.86E−02
	LOC105673464	Venom carboxylesterase‐6‐like (pseudo)	−0.88	6.57E−05	1.47E−02
	LOC105667368	46 kDa FK506‐binding nuclear protein‐like (LOC105667368)	−0.88	5.38E−04	6.26E−02
	LOC105667677	Zinc carboxypeptidase‐like (LOC105667677)	−0.88	6.17E−04	6.82E−02
	LOC105670134	Uncharacterized LOC105670134 (LOC105670134)	−0.88	5.08E−04	6.19E−02
	LOC105670241	Uncharacterized LOC105670241 (LOC105670241)	−0.89	3.70E−05	9.80E−03
	LOC105670430	Aminopeptidase N‐like (LOC105670430)	−0.91	5.43E−05	1.27E−02
	LOC105668482	Espin (LOC105668482)	−0.92	4.39E−04	5.60E−02
	LOC105670871	Uncharacterized LOC105670871 (LOC105670871)	−0.92	6.72E−04	7.06E−02
	LOC105677706	Bipolar kinesin KRP‐130‐like (LOC105677706)	−0.92	4.01E−04	5.24E−02
	LOC105678817	Protein toll‐like (LOC105678817)	−0.94	4.06E−05	1.04E−02
	LOC105674728	Muscle M‐line assembly protein unc‐89‐like (LOC105674728)	−0.94	1.14E−06	8.71E−04
	LOC105678912	Protein toll‐like (LOC105678912)	−0.94	8.07E−04	8.09E−02
	LOC105670239	Uncharacterized LOC105670239 (LOC105670239)	−0.98	4.27E−05	1.05E−02
	LOC105678342	Gephyrin‐like (LOC105678342)	−0.99	3.59E−04	4.87E−02
	LOC105678985	Uncharacterized LOC105678985 (LOC105678985)	−1.00	1.95E−05	6.35E−03
	LOC105670219	Uncharacterized LOC105670219 (LOC105670219)	−1.00	7.05E−04	7.13E−02
	LOC105674716	Zinc carboxypeptidase A 1‐like (LOC105674716)	−1.01	9.03E−05	1.90E−02
	LOC105670899	Uncharacterized LOC105670899 (LOC105670899)	−1.01	3.63E−05	9.80E−03
	LOC105678986	Uncharacterized LOC105678986 (LOC105678986)	−1.01	3.51E−04	4.83E−02
	LOC105678984	Uncharacterized LOC105678984 (LOC105678984)	−1.02	6.48E−06	3.31E−03
	LOC105667907	Thyroid receptor‐interacting protein 11‐like (LOC105667907)	−1.02	1.05E−03	9.99E−02
	LOC105669733	Venom carboxylesterase‐6‐like (LOC105669733)	−1.02	1.16E−04	2.14E−02
	LOC105678217	Uncharacterized LOC105678217 (LOC105678217)	−1.02	5.44E−05	1.27E−02
	LOC105671625	Uncharacterized LOC105671625 (LOC105671625)	−1.06	3.10E−05	8.75E−03
	LOC105676578	Thyrotropin‐releasing hormone‐degrading ectoenzyme‐like (LOC105676578)	−1.06	5.30E−04	6.25E−02
	LOC105678934	Uncharacterized LOC105678934 (LOC105678934)	−1.06	5.13E−04	6.19E−02
	LOC105678987	Uncharacterized LOC105678987 (LOC105678987)	−1.07	2.68E−04	3.99E−02
	LOC105675620	Aminopeptidase N‐like (LOC105675620)	−1.07	2.58E−04	3.96E−02
	LOC105674397	Uncharacterized LOC105674397 (LOC105674397)	−1.08	2.38E−05	7.32E−03
	LOC105670244	Uncharacterized LOC105670244 (LOC105670244)	−1.10	2.22E−04	3.56E−02
	LOC105670868	Uncharacterized LOC105670868 (LOC105670868)	−1.12	3.42E−06	1.93E−03
	LOC105674484	RCC1 and BTB domain‐containing protein 1‐like (LOC105674484)	−1.14	2.31E−04	3.59E−02
	LOC105676597	Uncharacterized LOC105676597 (LOC105676597)	−1.15	1.00E−04	2.03E−02
	LOC105674665	T‐box transcription factor TBX20‐like (LOC105674665)	−1.17	6.35E−04	6.89E−02
	LOC105674406	Uncharacterized LOC105674406 (LOC105674406)	−1.17	2.39E−05	7.32E−03
	LOC105679050	Transcription termination factor 2‐like (LOC105679050)	−1.19	2.05E−07	2.20E−04
	LOC105674677	Uncharacterized LOC105674677 (LOC105674677)	−1.20	1.10E−06	8.71E−04
	LOC105679162	Uncharacterized LOC105679162 (LOC105679162)	−1.21	1.39E−04	2.48E−02
	LOC105672421	Uncharacterized LOC105672421 (LOC105672421)	−1.22	1.02E−04	2.03E−02
	LOC105679916	Kinesin‐like protein KIF12 (LOC105679916)	−1.24	8.97E−06	4.01E−03
	LOC105675300	Aminopeptidase N‐like (LOC105675300)	−1.25	5.16E−04	6.19E−02
	LOC105672131	Uncharacterized LOC105672131 (LOC105672131)	−1.26	8.11E−05	1.74E−02
	LOC105678936	Uncharacterized LOC105678936 (LOC105678936)	−1.26	2.99E−04	4.30E−02
	LOC105670915	Probable salivary secreted peptide (LOC105670915)	−1.29	7.81E−11	1.40E−07
	LOC105676006	Uncharacterized LOC105676006 (LOC105676006)	−1.30	1.23E−05	5.08E−03
	LOC105668490	Uncharacterized LOC105668490 (LOC105668490)	−1.33	6.14E−04	6.82E−02
	LOC105678344	Gephyrin‐like (LOC105678344)	−1.33	1.92E−04	3.11E−02
	LOC105671608	Uncharacterized LOC105671608 (LOC105671608)	−1.35	2.62E−04	3.96E−02
	LOC105678353	Aminopeptidase N‐like (LOC105678353)	−1.43	2.18E−06	1.56E−03
	LOC105677450	Phosphotriesterase‐related protein‐like (LOC105677450)	−1.44	1.15E−04	2.14E−02
	LOC105670356	Sodium‐dependent nutrient amino acid transporter 1‐like (LOC105670356)	−1.45	1.64E−04	2.79E−02
	LOC105679178	Uncharacterized LOC105679178 (LOC105679178)	−1.45	5.80E−04	6.69E−02
	LOC105670873	Uncharacterized LOC105670873 (LOC105670873)	−1.45	4.31E−05	1.05E−02
	LOC105668011	Uncharacterized LOC105668011 (LOC105668011)	−1.51	2.29E−04	3.59E−02
	LOC105670875	Uncharacterized LOC105670875 (LOC105670875)	−1.53	6.10E−04	6.82E−02
	LOC105676970	A disintegrin and metalloproteinase with thrombospondin motifs 7‐like (LOC105676970)	−1.53	8.34E−04	8.28E−02
	LOC105679161	A disintegrin and metalloproteinase with thrombospondin motifs 17‐like (LOC105679161)	−1.54	3.75E−05	9.80E−03
	LOC105674012	Phosphotriesterase‐related protein‐like (LOC105674012)	−1.55	1.04E−04	2.03E−02
	LOC105676008	Uncharacterized LOC105676008 (LOC105676008)	−1.55	1.57E−05	6.03E−03
	LOC105679027	Uncharacterized LOC105679027 (LOC105679027)	−1.58	4.64E−04	5.85E−02
	LOC105674893	Uncharacterized LOC105674893 (LOC105674893)	−1.59	3.67E−04	4.89E−02
	LOC105673254	Thyrotropin‐releasing hormone‐degrading ectoenzyme‐like (LOC105673254)	−1.60	3.12E−04	4.40E−02
	LOC105674631	Uncharacterized LOC105674631 (LOC105674631)	−1.61	5.14E−04	6.19E−02
	LOC105671207	A disintegrin and metalloproteinase with thrombospondin motifs 7‐like (LOC105671207)	−1.63	2.47E−05	7.36E−03
	LOC105677676	Aminopeptidase N‐like (LOC105677676)	−1.67	3.69E−04	4.89E−02
	LOC105674526	Uncharacterized LOC105674526 (LOC105674526)	−1.71	8.17E−06	3.81E−03
	LOC105677348	Uncharacterized LOC105677348 (LOC105677348)	−1.73	2.80E−15	1.00E−11
	LOC105674527	Uncharacterized LOC105674527 (LOC105674527)	−1.74	2.66E−06	1.78E−03
	LOC105676143	A disintegrin and metalloproteinase with thrombospondin motifs 8‐like (LOC105676143)	−1.78	8.79E−04	8.65E−02
	LOC105677480	Uncharacterized LOC105677480 (LOC105677480)	−1.80	6.99E−04	7.13E−02
	LOC105678343	Gephyrin‐like (LOC105678343)	−1.86	5.09E−06	2.73E−03
	LOC105668553	A disintegrin and metalloproteinase with thrombospondin motifs 2‐like (LOC105668553)	−1.93	1.66E−04	2.79E−02
	LOC105673493	Alpha‐tocopherol transfer protein‐like (LOC105673493)	−1.94	6.81E−04	7.08E−02
	LOC105679148	Uncharacterized LOC105679148 (LOC105679148)	−1.99	1.59E−08	2.13E−05
	LOC105676228	Glutamyl aminopeptidase‐like (LOC105676228)	−2.02	3.17E−06	1.93E−03
	LOC105677332	Uncharacterized LOC105677332 (LOC105677332)	−2.05	2.87E−17	1.54E−13
	LOC105675423	Uncharacterized LOC105675423 (LOC105675423)	−2.08	1.51E−05	5.98E−03
	LOC105674732	Zinc carboxypeptidase‐like (LOC105674732)	−2.10	1.60E−04	2.76E−02
	LOC105674384	Uncharacterized LOC105674384 (LOC105674384)	−2.11	1.91E−05	6.35E−03
	LOC105675063	Alkaline phosphatase 4‐like (LOC105675063)	−2.13	1.79E−05	6.19E−03
	LOC105677347	Uncharacterized LOC105677347 (LOC105677347)	−2.16	9.71E−20	1.04E−15
	LOC105671515	A disintegrin and metalloproteinase with thrombospondin motifs 7‐like (LOC105671515)	−2.21	9.59E−06	4.11E−03
	LOC105677333	Uncharacterized LOC105677333 (LOC105677333)	−2.25	5.21E−14	1.40E−10
	LOC105667394	A disintegrin and metalloproteinase with thrombospondin motifs 12‐like (LOC105667394)	−2.30	1.37E−04	2.48E−02
	LOC105668554	A disintegrin and metalloproteinase with thrombospondin motifs 2‐like (LOC105668554)	−2.45	5.20E−04	6.19E−02
	LOC105679017	Uncharacterized LOC105679017 (LOC105679017)	−2.77	6.92E−05	1.51E−02
T4	LOC105679873	Sequestosome‐1 (LOC105679873)	3.08	3.32E−14	4.73E−11
	LOC105674352	Aromatic‐l‐amino acid decarboxylase (LOC105674352)	3.03	3.71E−13	4.39E−10
	LOC105667809	Protein lethal(2)essential for life‐like (LOC105667809)	3.02	1.13E−07	5.03E−05
	LOC105676758	Ninjurin‐1 (LOC105676758)	2.82	2.79E−07	1.10E−04
	LOC105678482	NF‐kappa‐B inhibitor cactus‐like (LOC105678482)	2.78	3.58E−09	2.12E−06
	LOC105678050	Uncharacterized LOC105678050 (LOC105678050)	2.64	8.90E−06	2.48E−03
	LOC105675162	Uncharacterized LOC105675162 (LOC105675162)	2.33	4.34E−06	1.40E−03
	LOC105679973	Fatty acid binding protein 1‐B.1‐like (LOC105679973)	1.97	1.70E−05	4.33E−03
	LOC105674105	Beta‐hexosaminidase subunit beta‐like (LOC105674105)	1.92	6.17E−05	1.19E−02
	LOC105678395	Uncharacterized LOC105678395 (LOC105678395)	1.89	6.75E−06	2.09E−03
	LOC105677178	Alpha‐2‐macroglobulin‐like protein 1 (LOC105677178) (TepII)	1.89	2.59E−06	9.12E−04
	LOC105671337	Putative fatty acyl‐CoA reductase CG5065 (LOC105671337)	1.86	2.04E−04	3.46E−02
	LOC105679392	Vanin‐like protein 1 (LOC105679392)	1.83	8.62E−06	2.48E−03
	LOC105677051	Protein Malvolio (LOC105677051)	1.80	2.15E−04	3.56E−02
	LOC105676443	Uncharacterized LOC105676443 (LOC105676443)	1.79	3.88E−05	8.35E−03
	LOC105679998	Heat shock 70 kDa protein cognate 4 (LOC105679998)	1.42	3.74E−04	5.42E−02
	LOC105669805	Facilitated trehalose transporter Tret1‐2 homolog (LOC105669805)	−1.56	7.81E−04	9.86E−02
	LOC105675281	Fibrillin‐1 (LOC105675281)	−1.67	4.31E−04	6.13E−02
	LOC105670714	Alpha‐amylase A (LOC105670714)	−1.78	4.73E−05	9.49E−03
	LOC105675053	Cytochrome P450 6a2‐like (LOC105675053)	−1.79	4.80E−04	6.57E−02
	LOC105667318	Dentin sialophosphoprotein‐like (LOC105667318)	−1.87	2.69E−05	6.21E−03
	LOC105676465	Facilitated trehalose transporter Tret1 (LOC105676465)	−1.87	3.55E−04	5.26E−02
	LOC105676200	Short‐chain dehydrogenase/reductase family 16C member 6‐like (LOC105676200)	−1.88	2.75E−04	4.45E−02
	LOC105676829	Stearoyl‐CoA desaturase 5‐like (LOC105676829)	−1.90	6.18E−04	8.14E−02
	LOC105669660	Nose resistant to fluoxetine protein 6‐like (LOC105669660)	−1.97	7.40E−04	9.57E−02
	LOC105673315	Pheromone‐binding protein Gp‐9‐like (LOC105673315)	−2.04	2.93E−04	4.64E−02
	LOC105677462	Peroxisomal hydratase‐dehydrogenase‐epimerase‐like (LOC105677462)	−2.13	3.14E−04	4.85E−02
	LOC105679763	Uncharacterized oxidoreductase C26H5.09c‐like (LOC105679763)	−2.15	9.02E−05	1.67E−02
	LOC105672608	Facilitated trehalose transporter Tret1‐like (LOC105672608)	−2.26	1.92E−04	3.32E−02
	LOC105671746	Probable cytochrome P450 304a1 (LOC105671746)	−2.31	6.96E−07	2.61E−04
	LOC105677950	Elongation of very long chain fatty acids protein 4‐like (LOC105677950)	−2.51	4.67E−04	6.52E−02
	LOC105670857	Leucine‐rich repeat‐containing protein egg‐6 (LOC105670857)	−2.53	1.57E−05	4.14E−03
	LOC105671912	Vitellogenin‐1‐like (LOC105671912)	−2.73	5.67E−04	7.62E−02
	LOC105678691	Scavenger receptor class B member 1‐like (LOC105678691)	−2.75	5.51E−09	3.02E−06
	LOC105676041	Uncharacterized LOC105676041 (LOC105676041)	−2.80	4.87E−11	3.85E−08
	LOC105674885	Purine nucleoside phosphorylase‐like (LOC105674885)	−2.83	9.06E−06	2.48E−03
	LOC105674435	Serine protease inhibitor dipetalogastin‐like (LOC105674435)	−2.84	2.69E−06	9.12E−04
	LOC105677195	Glucose dehydrogenase [FAD	−2.85	2.71E−05	6.21E−03
	LOC105669773	Regucalcin‐like (LOC105669773)	−2.85	1.81E−08	8.60E−06
	LOC105668188	Uncharacterized LOC105668188 (LOC105668188)	−2.86	1.41E−08	7.16E−06
	LOC105674040	Apolipophorins (LOC105674040)	−2.88	1.39E−07	5.80E−05
	LOC105679486	Acyl‐CoA Delta(11) desaturase‐like (LOC105679486)	−2.92	7.97E−04	9.86E−02
	LOC105669905	Uncharacterized LOC105669905 (LOC105669905)	−2.95	9.17E−05	1.67E−02
	LOC105675057	Uncharacterized LOC105675057 (LOC105675057)	−2.95	3.30E−04	4.99E−02
	LOC105672219	Leucine‐rich repeat‐containing protein 4‐like (LOC105672219)	−2.98	2.37E−05	5.82E−03
	LOC105670441	Uncharacterized LOC105670441 (LOC105670441)	−3.07	1.75E−04	3.11E−02
	LOC105672550	Uncharacterized LOC105672550 (LOC105672550)	−3.13	4.27E−12	4.34E−09
	LOC105670856	Negative regulator of reactive oxygen species‐like (LOC105670856)	−3.21	2.74E−09	1.77E−06
	LOC105675773	Peptidoglycan‐recognition protein SC2‐like (LOC105675773)	−3.24	3.51E−15	6.23E−12
	LOC105678491	Uncharacterized LOC105678491 (LOC105678491)	−3.67	9.17E−12	8.16E−09
	LOC105671873	Uncharacterized LOC105671873 (LOC105671873)	−3.69	1.17E−20	2.78E−17
	LOC105677373	Probable WRKY transcription factor protein 1 (LOC105677373)	−4.04	2.11E−09	1.50E−06
	LOC105667550	Uncharacterized LOC105667550 (LOC105667550)	−4.95	4.11E−05	8.60E−03
	LOC105671872	Vitellogenin‐2‐like (LOC105671872)	−5.08	2.66E−36	1.89E−32
	LOC105669522	Glucose dehydrogenase [FAD	−5.08	2.89E−05	6.42E−03
	LOC105670022	Chymotrypsin‐2‐like (LOC105670022)	−5.18	1.02E−27	3.64E−24
	LOC105668189	Uncharacterized LOC105668189 (LOC105668189)	−11.64	4.80E−05	9.49E−03
	LOC105670097	Spidroin‐1 (LOC105670097)	−13.25	8.03E−04	9.86E−02
T8	LOC105669806	Cytochrome P450 307a1‐like (LOC105669806)	1.36	1.15E−07	4.64E−04
	LOC105676242	Uncharacterized LOC105676242 (LOC105676242)	1.31	1.03E−05	1.19E−02
	LOC105671866	Serine protease gd‐like (LOC105671866)	1.31	5.74E−06	9.27E−03
	LOC105672126	Uncharacterized LOC105672126 (LOC105672126)	1.09	7.45E−05	6.35E−02
	LOC105671608	Uncharacterized LOC105671608 (LOC105671608)	1.06	1.72E−04	9.96E−02
	LOC105674427	Beta‐1,3‐glucan‐binding protein‐like (LOC105674427)	1.02	7.86E−05	6.35E−02
	LOC105668572	Uncharacterized LOC105668572 (LOC105668572)	0.91	4.88E−08	3.94E−04
	LOC105668988	Uncharacterized LOC105668988 (LOC105668988)	0.90	1.43E−04	9.96E−02
	LOC105677178	Alpha‐2‐macroglobulin‐like protein 1 (LOC105677178) (TepII)	0.73	1.63E−04	9.96E−02
	LOC105675407	Inhibin beta E chain (LOC105675407)	−0.77	7.26E−05	6.35E−02
	LOC105677462	Peroxisomal hydratase‐dehydrogenase‐epimerase‐like (LOC105677462)	−0.89	1.54E−04	9.96E−02
	LOC105672608	Facilitated trehalose transporter Tret1‐like (LOC105672608)	−0.93	7.57E−06	1.02E−02
	LOC105676062	Protein henna (LOC105676062)	−1.30	4.41E−06	8.90E−03
	LOC105668757	Uncharacterized LOC105668757 (LOC105668757)	−2.03	4.17E−07	1.12E−03
T12	LOC105668184	Uncharacterized LOC105668184 (LOC105668184)	8.58	1.64E−08	8.01E−06
	LOC105675287	Major royal jelly protein 1 (LOC105675287)	5.05	1.72E−29	6.14E−26
	LOC105670097	Spidroin‐1 (LOC105670097)	3.47	4.32E−04	5.14E−02
	LOC105678813	Uncharacterized LOC105678813 (LOC105678813)	3.45	6.53E−43	3.50E−39
	LOC105669245	Ejaculatory bulb‐specific protein 3‐like (LOC105669245)	2.92	5.39E−06	1.41E−03
	LOC105676540	Uncharacterized LOC105676540 (LOC105676540)	2.86	7.75E−29	2.08E−25
	LOC105676242	Uncharacterized LOC105676242 (LOC105676242)	2.63	3.00E−23	6.44E−20
	LOC105670707	Uncharacterized transmembrane protein DDB_G0289901‐like (LOC105670707)	2.17	5.77E−45	6.19E−41
	LOC105679181	Facilitated trehalose transporter Tret1 (LOC105679181)	1.87	1.22E−09	6.87E−07
	LOC105672089	Lipid storage droplets surface‐binding protein 2‐like (LOC105672089)	1.87	1.94E−09	1.04E−06
	LOC105671866	Serine protease gd‐like (LOC105671866)	1.70	4.82E−11	3.44E−08
	LOC105672123	Uncharacterized LOC105672123 (LOC105672123)	1.63	3.02E−05	5.73E−03
	LOC105674930	Facilitated trehalose transporter Tret1‐like (LOC105674930)	1.60	2.18E−07	8.67E−05
	LOC105673362	Limulus clotting factor C‐like (LOC105673362)	1.55	6.18E−07	2.14E−04
	LOC105669469	Cytochrome b5‐like (LOC105669469)	1.46	6.81E−13	7.30E−10
	LOC105675162	Uncharacterized LOC105675162 (LOC105675162)	1.46	1.35E−16	2.41E−13
	LOC105677051	Protein Malvolio (LOC105677051)	1.44	3.83E−12	3.42E−09
	LOC105676758	Ninjurin‐1 (LOC105676758)	1.41	1.24E−07	5.10E−05
	LOC105678482	NF‐kappa‐B inhibitor cactus‐like (LOC105678482)	1.40	8.63E−16	1.32E−12
	LOC105678075	Tyrosine 3‐monooxygenase (LOC105678075)	1.40	1.51E−13	1.80E−10
	LOC105673363	Limulus clotting factor C‐like (LOC105673363)	1.37	3.02E−09	1.54E−06
	LOC105675429	Cyclic GMP‐AMP synthase (LOC105675429)	1.29	5.14E−08	2.29E−05
	LOC105677645	Cytochrome P450 6A1‐like (LOC105677645)	1.24	9.63E−05	1.54E−02
	LOC105677207	Gamma‐glutamyl transpeptidase 1‐like (LOC105677207)	1.21	4.40E−12	3.63E−09
	LOC105679973	Fatty acid binding protein 1‐B.1‐like (LOC105679973)	1.21	3.53E−06	1.02E−03
	LOC105676748	Cysteine sulfinic acid decarboxylase (LOC105676748)	1.19	1.21E−06	3.92E−04
	LOC105671174	Lipopolysaccharide‐induced tumor necrosis factor‐alpha factor homolog (LOC105671174)	1.15	2.95E−06	8.78E−04
	LOC105672439	Alpha‐sarcoglycan (LOC105672439)	1.15	6.89E−08	2.95E−05
	LOC105679722	Uncharacterized LOC105679722 (LOC105679722)	1.12	5.34E−04	6.10E−02
	LOC105678784	Protein toll‐like (LOC105678784)	1.11	3.29E−04	4.15E−02
	LOC105671850	Protein‐S‐isoprenylcysteine O‐methyltransferase (LOC105671850)	1.10	1.57E−05	3.11E−03
	LOC105677622	Uncharacterized LOC105677622 (LOC105677622)	1.08	2.16E−06	6.61E−04
	LOC105677585	Venom protease‐like (LOC105677585)	1.06	1.20E−05	2.48E−03
	LOC105673874	2‐oxoisovalerate dehydrogenase subunit beta	1.03	7.40E−05	1.24E−02
	LOC105672119	Glutamine:fructose‐6‐phosphate aminotransferase [isomerizing] 2‐like (LOC105672119)	1.03	4.34E−07	1.55E−04
	LOC105677178	Alpha‐2‐macroglobulin‐like protein 1 (LOC105677178) (TepII)	1.02	2.65E−07	1.02E−04
	LOC105672744	Probable phospholipid‐transporting ATPase VD (LOC105672744)	0.99	3.88E−06	1.09E−03
	LOC105667656	Uncharacterized LOC105667656 (LOC105667656)	0.98	1.82E−05	3.54E−03
	LOC105678551	Uncharacterized LOC105678551 (LOC105678551)	0.96	4.23E−04	5.12E−02
	LOC105676247	Uncharacterized LOC105676247 (LOC105676247)	0.95	8.77E−06	2.09E−03
	LOC105671291	Putative inorganic phosphate cotransporter (LOC105671291)	0.92	8.11E−04	8.44E−02
	LOC105673744	Uncharacterized LOC105673744 (LOC105673744) (Serpin‐27A‐like)	0.91	1.01E−04	1.60E−02
	LOC105672065	Uncharacterized LOC105672065 (LOC105672065)	0.91	3.06E−04	4.05E−02
	LOC105677172	Protein dimmed‐like (LOC105677172)	0.90	9.81E−06	2.19E−03
	LOC105675116	Sterol 24‐C‐methyltransferase‐like (LOC105675116)	0.89	5.33E−05	9.08E−03
	LOC105668572	Uncharacterized LOC105668572 (LOC105668572)	0.89	1.39E−04	2.09E−02
	LOC105672359	Thiamine transporter 2‐like (LOC105672359)	0.88	7.74E−05	1.28E−02
	LOC105673919	ATP‐binding cassette sub‐family C member 9 (LOC105673919)	0.88	3.12E−05	5.78E−03
	LOC105668941	Probable citrate synthase 2, mitochondrial (LOC105668941)	0.86	3.49E−05	6.23E−03
	LOC105669588	Apoptosis‐inducing factor 3 (LOC105669588)	0.85	2.78E−07	1.03E−04
	LOC105674532	ETS homologous factor‐like (LOC105674532)	0.84	3.01E−04	4.05E−02
	LOC105678679	AN1‐type zinc finger protein 2A‐like (LOC105678679)	0.84	5.66E−04	6.38E−02
	LOC105677494	Annexin B9 (LOC105677494)	0.83	7.92E−06	1.97E−03
	LOC105670266	Sodium/potassium/calcium exchanger 3‐like (LOC105670266)	0.81	9.60E−06	2.19E−03
	LOC105679962	Thioredoxin reductase 1, mitochondrial (LOC105679962)	0.81	5.39E−06	1.41E−03
	LOC105668729	Nuclear factor NF‐kappa‐B p100 subunit (LOC105668729) (Relish)	0.78	3.01E−04	4.05E−02
	LOC105678483	NF‐kappa‐B inhibitor cactus‐like (LOC105678483)	0.73	4.25E−04	5.12E−02
	LOC105675225	Kelch‐like ECH‐associated protein 1 (LOC105675225)	0.71	1.28E−04	1.96E−02
	LOC105678890	Sodium‐independent sulfate anion transporter (LOC105678890)	0.71	7.95E−04	8.44E−02
	LOC105673596	Putative inorganic phosphate cotransporter (LOC105673596)	0.62	4.03E−04	5.03E−02
	LOC105675851	Sphingosine kinase 2 (LOC105675851)	0.62	7.01E−04	7.66E−02
	LOC105677558	Kinectin‐like (LOC105677558)	0.61	1.64E−04	2.40E−02
	LOC105668823	Uncharacterized LOC105668823 (LOC105668823)	0.61	9.88E−04	9.77E−02
	LOC105674043	Uncharacterized LOC105674043 (LOC105674043)	0.60	2.51E−04	3.54E−02
	LOC105671136	Serine protease 52‐like (LOC105671136)	0.53	8.59E−04	8.85E−02
	LOC105678761	Serine protease inhibitor 3/4‐like (LOC105678761)	−0.51	9.14E−04	9.33E−02
	LOC105672958	Cytochrome P450 9e2‐like (LOC105672958)	−0.67	1.01E−03	9.87E−02
	LOC105668778	Leucine‐rich repeats and immunoglobulin‐like domains protein 2 (LOC105668778)	−0.67	9.81E−04	9.77E−02
	LOC105671541	Protein croquemort‐like (LOC105671541)	−0.68	9.94E−04	9.77E−02
	LOC105676100	Tubulin‐specific chaperone cofactor E‐like protein (LOC105676100)	−0.71	5.35E−04	6.10E−02
	LOC105667678	Monocarboxylate transporter 12‐B (LOC105667678)	−0.75	6.13E−04	6.85E−02
	LOC105672522	Rho‐related BTB domain‐containing protein 1 (LOC105672522)	−0.76	6.84E−04	7.56E−02
	LOC105677283	Alpha‐aminoadipic semialdehyde synthase (LOC105677283)	−0.77	1.62E−04	2.40E−02
	LOC105673034	PI‐PLC × domain‐containing protein 1‐like (LOC105673034)	−0.78	8.02E−04	8.44E−02
	LOC105677249	Glycogen‐binding subunit 76A (LOC105677249)	−0.79	9.57E−05	1.54E−02
	LOC105667709	Uncharacterized LOC105667709 (LOC105667709)	−0.79	4.18E−04	5.12E−02
	LOC105680137	Tryptophan 2	−0.79	4.02E−05	7.07E−03
	LOC105674346	Neural‐cadherin (LOC105674346)	−0.83	3.04E−04	4.05E−02
	LOC105670852	Steroid receptor seven‐up, isoforms B/C (LOC105670852)	−0.84	1.99E−04	2.84E−02
	LOC105678750	Uncharacterized LOC105678750 (LOC105678750)	−0.85	4.64E−04	5.41E−02
	LOC105669560	Inositol oxygenase (LOC105669560)	−0.85	8.16E−06	1.99E−03
	LOC105668188	Uncharacterized LOC105668188 (LOC105668188)	−0.85	8.11E−04	8.44E−02
	LOC105669693	Aryl hydrocarbon receptor nuclear translocator‐like protein 1 (LOC105669693)	−0.87	1.70E−04	2.46E−02
	LOC105669509	Arylsulfatase B‐like (LOC105669509)	−0.92	4.77E−05	8.24E−03
	LOC105679739	Uncharacterized LOC105679739 (LOC105679739)	−0.96	5.10E−08	2.29E−05
	LOC105672956	Ribose‐phosphate pyrophosphokinase 1 (LOC105672956)	−0.98	7.94E−04	8.44E−02
	LOC105680118	Cysteine proteinase 1‐like (LOC105680118)	−0.99	3.23E−04	4.12E−02
	LOC105677063	Organic cation transporter protein (LOC105677063)	−1.01	1.51E−05	3.06E−03
	LOC105674681	Cytochrome P450 6j1‐like (LOC105674681)	−1.03	3.11E−04	4.05E−02
	LOC105672502	Alpha‐tocopherol transfer protein‐like (LOC105672502)	−1.03	3.13E−04	4.05E−02
	LOC105671942	Hexokinase‐2‐like (LOC105671942)	−1.04	1.48E−10	9.89E−08
	LOC105667318	Dentin sialophosphoprotein‐like (LOC105667318)	−1.04	3.05E−05	5.73E−03
	LOC105680018	Uncharacterized LOC105680018 (LOC105680018)	−1.11	1.16E−05	2.43E−03
	LOC105667320	Homeobox protein Nkx‐2.2a‐like (LOC105667320)	−1.16	9.78E−04	9.77E−02
	LOC105671469	d‐arabinitol dehydrogenase 1‐like (LOC105671469)	−1.17	2.77E−04	3.86E−02
	LOC105671862	Uncharacterized LOC105671862 (LOC105671862)	−1.17	3.35E−05	6.08E−03
	LOC105677265	Tachykinin‐like peptides receptor 99D (LOC105677265)	−1.25	6.72E−07	2.25E−04
	LOC105671615	Zinc finger protein 76‐like (LOC105671615)	−1.26	1.12E−05	2.43E−03
	LOC105670688	Uncharacterized LOC105670688 (LOC105670688)	−1.32	4.53E−04	5.33E−02
	LOC105678416	Pancreatic lipase‐related protein 2‐like (LOC105678416)	−1.36	1.73E−11	1.32E−08
	LOC105676634	Carboxypeptidase B‐like (LOC105676634)	−1.39	1.55E−06	4.88E−04
	LOC105668871	Phenoloxidase 2‐like (LOC105668871)	−1.42	4.69E−06	1.29E−03
	LOC105679484	Sorbitol dehydrogenase‐like (LOC105679484)	−1.45	1.09E−04	1.70E−02
	LOC105675407	Inhibin beta E chain (LOC105675407)	−1.57	1.85E−15	2.47E−12
	LOC105670471	Trypsin epsilon‐like (LOC105670471)	−1.64	1.15E−05	2.43E−03
	LOC105678258	Facilitated trehalose transporter Tret1‐like (LOC105678258)	−1.87	6.36E−06	1.62E−03
	LOC105672897	Cytochrome P450 4C1‐like (pseudo)	−1.97	1.08E−09	6.45E−07
	LOC105672608	Facilitated trehalose transporter Tret1‐like (LOC105672608)	−2.18	1.63E−10	1.03E−07
	LOC105672083	Cytochrome P450 4c21‐like (LOC105672083)	−2.42	9.57E−06	2.19E−03
	LOC105677462	Peroxisomal hydratase‐dehydrogenase‐epimerase‐like (LOC105677462)	−2.45	2.17E−12	2.11E−09

LFC: log_2_ fold change.

## References

[ece34573-bib-0001] Adamo, S. A. (2017). The stress response and immune system share, borrow, and reconfigure their physiological network elements: Evidence from the insects. Hormones and Behavior, 88, 25–30. 10.1016/j.yhbeh.2016.10.003 27746212

[ece34573-bib-0002] Al‐Shahrour, F. , Diaz‐Uriarte, R. , & Dopazo, J. (2004). FatiGO: A web tool for finding significant associations of gene ontology terms with groups of genes. Bioinformatics, 20(4), 578–580. 10.1093/bioinformatics/btg455 14990455

[ece34573-bib-0003] Anders, S. , Pyl, P. T. , & Huber, W. (2015). HTSeq—A python framework to work with high‐throughput sequencing data. Bioinformatics, 31(2), 166–169. 10.1093/bioinformatics/btu638 25260700 PMC4287950

[ece34573-bib-0004] Aubert, A. , & Richard, F. J. (2008). Social management of LPS‐induced inflammation in *Formica polyctena* ants. Brain, Behavior, and Immunity, 22(6), 833–837. 10.1016/j.bbi.2008.01.010 18331785

[ece34573-bib-0005] Barribeau, S. M. , Sadd, B. M. , Du Plessis, L. , Brown, M. J. , Buechel, S. D. , Cappelle, K. , … Schmid‐Hempel, P. (2015). A depauperate immune repertoire precedes evolution of sociality in bees. Genome Biology, 16(1), 83 10.1186/s13059-015-0628-y 25908406 PMC4408586

[ece34573-bib-0006] Benjamini, Y. , & Hochberg, Y. (1995). Controlling the false discovery rate: A practical and powerful approach to multiple testing. Journal of the Royal Statistical Society. Series B (Methodological), 57(1), 289–300.

[ece34573-bib-0007] Bischoff, V. , Vignal, C. , Duvic, B. , Boneca, I. G. , Hoffmann, J. A. , & Royet, J. (2006). Downregulation of the *Drosophila* immune response by peptidoglycan‐recognition proteins SC1 and SC2. PLoS Pathogens, 2(2), e14 10.1371/journal.ppat.0020014 16518472 PMC1383489

[ece34573-bib-0008] Borba, R. S. , Klyczek, K. K. , Mogen, K. L. , & Spivak, M. (2015). Seasonal benefits of a natural propolis envelope to honey bee immunity and colony health. Journal of Experimental Biology, 218(22), 3689–3699. 10.1242/jeb.127324 26449975

[ece34573-bib-0009] Boulay, R. , Quagebeur, M. , Godzinska, E. , & Lenoir, A. (1999). Social isolation in ants: Evidence of its impact on survivorship and behavior in *Camponotus fellah* (Hymenoptera: Formicidae). Sociobiology, 33(2), 111–124.

[ece34573-bib-0010] Bourke, A. F. G. , & Franks, N. R. (1995). Social evolution in ants. Princeton, NJ: Princeton University Press.

[ece34573-bib-0011] Buchon, N. , Poidevin, M. , Kwon, H. , Guillou, A. , Sottas, V. , Lee, B. , & Lemaitre, B. (2009). A single modular serine protease integrates signals from pattern‐recognition receptors upstream of the *Drosophila* Toll pathway. Proceedings of the National Academy of Sciences of the United States of America, 106(30), 12442–12447. 10.1073/pnas.0901924106 19590012 PMC2718337

[ece34573-bib-0012] Casteels, P. , Ampe, C. , Jacobs, F. , & Tempst, P. (1993). Functional and chemical characterization of Hymenoptaecin, an antibacterial polypeptide that is infection‐inducible in the honeybee (*Apis mellifera*). Journal of Biological Chemistry, 268(10), 7044–7054. 8463238

[ece34573-bib-0013] Castella, G. , Chapuisat, M. , Moret, Y. , & Christe, P. (2008). The presence of conifer resin decreases the use of the immune system in wood ants. Ecological Entomology, 33(3), 408–412. 10.1111/j.1365-2311.2007.00983.x

[ece34573-bib-0014] Chapuisat, M. , Oppliger, A. , Magliano, P. , & Christe, P. (2007). Wood ants use resin to protect themselves against pathogens. Proceedings of the Royal Society B: Biological Sciences, 274(1621), 2013–2017. 17535794 10.1098/rspb.2007.0531 PMC2275180

[ece34573-bib-0015] Choo, Y. M. , Lee, K. S. , Yoon, H. J. , Kim, B. Y. , Sohn, M. R. , Roh, J. Y. , … Jin, B. R. (2010). Dual function of a bee venom serine protease: Prophenoloxidase‐activating factor in arthropods and fibrin(ogen)olytic enzyme in mammals. PLoS One, 5(5), e10393 10.1371/journal.pone.0010393 20454652 PMC2862700

[ece34573-bib-0016] Christe, P. , Oppliger, A. , Bancalà, F. , Castella, G. , & Chapuisat, M. (2003). Evidence for collective medication in ants. Ecology Letters, 6(168), 19–22. 10.1046/j.1461-0248.2003.00395.x

[ece34573-bib-0017] Conesa, A. , Gotz, S. , Garcia‐Gomez, J. M. , Terol, J. , Talon, M. , & Robles, M. (2005). Blast2GO: A universal tool for annotation, visualization and analysis in functional genomics research. Bioinformatics, 21(18), 3674–3676. 10.1093/bioinformatics/bti610 16081474

[ece34573-bib-0018] Cremer, S. , Armitage, S. A. , & Schmid‐Hempel, P. (2007). Social immunity. Current Biology, 17(16), R693–R702. 10.1016/j.cub.2007.06.008 17714663

[ece34573-bib-0019] Cremer, S. , Pull, C. D. , & Fürst, M. A. (2018). Social immunity: Emergence and evolution of colony‐level disease protection. Annual Review of Entomology, 63, 105–123. 10.1146/annurev-ento-020117-043110 28945976

[ece34573-bib-0020] De Gregorio, E. , Han, S. , Lee, W. , Baek, M. , Osaki, T. , Kawabata, S. , … Brey, P. T. (2002). An immune‐responsive serpin regulates the melanization cascade in *Drosophila* . Developmental Cell, 3(4), 581–592. 10.1016/S1534-5807(02)00267-8 12408809

[ece34573-bib-0021] Dobin, A. , & Gingeras, T. R. (2015). Mapping RNA‐seq reads with STAR. Current Protocols in Bioinformatics, 51, 11.14.1–11.14.19. 26334920 10.1002/0471250953.bi1114s51 PMC4631051

[ece34573-bib-0022] Doublet, V. , Poeschl, Y. , Gogol‐Döring, A. , Alaux, C. , Annoscia, D. , Aurori, C. , … Grozinger, C. M. (2017). Unity in defence: Honeybee workers exhibit conserved molecular responses to diverse pathogens. BMC Genomics, 18(1), 207 10.1186/s12864-017-3597-6 28249569 PMC5333379

[ece34573-bib-0023] Erler, S. , Popp, M. , & Lattorff, H. M. G. (2011). Dynamics of immune system gene expression upon bacterial challenge and wounding in a social insect (*Bombus terrestris*). PLoS One, 6(3), e18126 10.1371/journal.pone.0018126 21479237 PMC3066223

[ece34573-bib-0024] Evans, J. D. , Aronstein, K. , Chen, Y. P. , Hetru, C. , Imler, J. L. , Jiang, H. , … Hultmark, D. (2006). Immune pathways and defence mechanisms in honey bees *Apis mellifera* . Insect Molecular Biology, 15(5), 645–656. 10.1111/j.1365-2583.2006.00682.x 17069638 PMC1847501

[ece34573-bib-0025] Evans, J. D. , & Spivak, M. (2010). Socialized medicine: Individual and communal disease barriers in honey bees. Journal of Invertebrate Pathology, 103, S62–S72. 10.1016/j.jip.2009.06.019 19909975

[ece34573-bib-0026] Ferrandon, D. , Imler, J. L. , Hetru, C. , & Hoffmann, J. A. (2007). The *Drosophila* systemic immune response: Sensing and signalling during bacterial and fungal infections. Nature Reviews Immunology, 7(11), 862–874. 10.1038/nri2194 17948019

[ece34573-bib-0027] Fogaça, A. C. , Almeida, I. C. , Eberlin, M. N. , Tanaka, A. S. , Bulet, P. , & Daffre, S. (2006). Ixodidin, a novel antimicrobial peptide from the hemocytes of the cattle tick *Boophilus microplus* with inhibitory activity against serine proteinases. Peptides, 27(4), 667–674. 10.1016/j.peptides.2005.07.013 16191451

[ece34573-bib-0028] Gerth, M. , & Hurst, G. D. (2017). Short reads from honey bee (*Apis* sp.) sequencing projects reflect microbial associate diversity. PeerJ, 5, e3529. 28717593 10.7717/peerj.3529 PMC5510586

[ece34573-bib-0029] Gruber, M. A. M. , Cooling, M. , Baty, J. W. , Buckley, K. , Friedlander, A. , Quinn, O. , … Lester, P. J. (2017). Single‐stranded RNA viruses infecting the invasive Argentine ant, *Linepithema humile* . Scientific Reports, 7(1), 3304 10.1038/s41598-017-03508-z 28607437 PMC5468335

[ece34573-bib-0030] Gubb, D. , Sanz‐Parra, A. , Barcena, L. , Troxler, L. , & Fullaondo, A. (2010). Protease inhibitors and proteolytic signalling cascades in insects. Biochimie, 92(12), 1749–1759. 10.1016/j.biochi.2010.09.004 20850496

[ece34573-bib-0031] Guillou, A. , Troha, K. , Wang, H. , Franc, N. C. , & Buchon, N. (2016). The *Drosophila* CD36 homologue *croquemort* is required to maintain immune and gut homeostasis during development and aging. PLoS Pathogens, 12(10), e1005961 10.1371/journal.ppat.1005961 27780230 PMC5079587

[ece34573-bib-0033] Hernández López, J. , Riessberger‐Gallé, U. , Crailsheim, K. , & Schuehly, W. (2017). Cuticular hydrocarbon cues of immune‐challenged workers elicit immune activation in honeybee queens. Molecular Ecology, 26(11), 3062–3073. 10.1111/mec.14086 28271576

[ece34573-bib-0034] Howard, R. W. , & Blomquist, G. J. (2005). Ecological, behavioral, and biochemical aspects of insect hydrocarbons. Annual Review of Entomology, 50, 371–393. 10.1146/annurev.ento.50.071803.130359 15355247

[ece34573-bib-0035] Hughes, W. O. H. , Eilenberg, J. , & Boomsma, J. J. (2002). Trade‐offs in group living: Transmission and disease resistance in leaf‐cutting ants. Proceedings of the Royal Society B: Biological Sciences, 269(1502), 1811–1819. 12350269 10.1098/rspb.2002.2113 PMC1691100

[ece34573-bib-0036] Jones, P. , Binns, D. , Chang, H. , Fraser, M. , Li, W. , McAnulla, C. , … Hunter, S. (2014). InterProScan 5: Genome‐scale protein function classification. Bioinformatics, 30(9), 1236–1240. 10.1093/bioinformatics/btu031 24451626 PMC3998142

[ece34573-bib-0037] Kleino, A. , Myllymäki, H. , Kallio, J. , Vanha‐aho, L. M. , Oksanen, K. , Ulvila, J. , … Ramet, M. (2008). Pirk is a negative regulator of the *Drosophila* Imd pathway. Journal of Immunology, 180(8), 5413–5422. 10.4049/jimmunol.180.8.5413 18390723

[ece34573-bib-0038] Kocks, C. , Cho, J. H. , Nehme, N. , Ulvila, J. , Pearson, A. M. , Meister, M. , … Ezekowitz, R. A. B. (2005). Eater, a transmembrane protein mediating phagocytosis of bacterial pathogens in *Drosophila* . Cell, 123(2), 335–346. 10.1016/j.cell.2005.08.034 16239149

[ece34573-bib-0039] Kohlmeier, P. , Holländer, K. , & Meunier, J. (2016). Survival after pathogen exposure in group‐living insects: Don't forget the stress of social isolation!. Journal of Evolutionary Biology, 29(9), 1867–1872. 10.1111/jeb.12916 27272199

[ece34573-bib-0041] Koto, A. , Mersch, D. , Hollis, B. , & Keller, L. (2015). Social isolation causes mortality by disrupting energy homeostasis in ants. Behavioral Ecology and Sociobiology, 69(4), 583–591. 10.1007/s00265-014-1869-6

[ece34573-bib-0042] Li, H. , & Durbin, R. (2009). Fast and accurate short read alignment with burrows‐wheeler transform. Bioinformatics, 25(14), 1754–1760. 10.1093/bioinformatics/btp324 19451168 PMC2705234

[ece34573-bib-0043] Li, H. , Handsaker, B. , Wysoker, A. , Fennell, T. , Ruan, J. , Homer, N. , … Durbin, R. (2009). The sequence Alignment/Map format and SAMtools. Bioinformatics, 25(16), 2078–2079. 10.1093/bioinformatics/btp352 19505943 PMC2723002

[ece34573-bib-0044] Love, M. I. , Huber, W. , & Anders, S. (2014). Moderated estimation of fold change and dispersion for RNA‐seq data with DESeq2. Genome Biology, 15(12), 1–21. 10.1186/s13059-014-0550-8 PMC4302049 25516281

[ece34573-bib-0045] Maki, K. , Shibata, T. , & Kawabata, S. I. (2017). Transglutaminase‐catalyzed incorporation of polyamines masks the DNA‐binding region of the transcription factor Relish. The Journal of Biological Chemistry, 292(15), 6369–6380. 10.1074/jbc.M117.779579 28258224 PMC5391764

[ece34573-bib-0046] NCBI Resource Coordinators (2017). Database resources of the national center for biotechnology information. Nucleic Acids Research, 45(Database, issue), D12–D17. 27899561 10.1093/nar/gkw1071 PMC5210554

[ece34573-bib-0047] Pili‐Floury, S. , Leulier, F. , Takahashi, K. , Saigo, K. , Samain, E. , Ueda, R. , & Lemaitre, B. (2004). *In vivo* RNA interference analysis reveals an unexpected role for GNBP1 in the defense against Gram‐positive bacterial infection in *Drosophila* adults. The Journal of Biological Chemistry, 279(13), 12848–12853. 14722090 10.1074/jbc.M313324200

[ece34573-bib-0048] Price, M. N. , & Arkin, A. P. (2017). PaperBLAST: Text mining papers for information about homologs. mSystems, 2(4), e00039–17 10.1128/mSystems.00039-17 28845458 PMC5557654

[ece34573-bib-0049] Pull, C. D. , Ugelvig, L. V. , Wiesenhofer, F. , Grasse, A. V. , Tragust, S. , Schmitt, T. , … Cremer, S. (2018). Destructive disinfection of infected brood prevents systemic disease spread in ant colonies. eLife, 7, e32073 10.7554/eLife.32073 29310753 PMC5760203

[ece34573-bib-0050] R Core Team (2015). R: A language and environment for statistical computing. Vienna, Austria: R foundation for statistical computing Retrieved from https://www.R-project.org/

[ece34573-bib-0051] Richard, F. , Holt, H. L. , & Grozinger, C. M. (2012). Effects of immunostimulation on social behavior, chemical communication and genome‐wide gene expression in honey bee workers (*Apis mellifera*). BMC Genomics, 13, 558 10.1186/1471-2164-13-558 23072398 PMC3483235

[ece34573-bib-0052] Richter, J. , Helbing, S. , Erler, S. , & Lattorff, H. M. G. (2012). Social context‐dependent immune gene expression in bumblebees (*Bombus terrestris*). Behavioral Ecology and Sociobiology, 66(5), 791–796. 10.1007/s00265-012-1327-2

[ece34573-bib-0053] Riedel, F. , Vorkel, D. , & Eaton, S. (2011). Megalin‐dependent yellow endocytosis restricts melanization in the *Drosophila* cuticle. Development, 138(1), 149–158. 10.1242/dev.056309 21138977

[ece34573-bib-0054] Rose, T. , LeMosy, E. K. , Cantwell, A. M. , Banerjee‐Roy, D. , Skeath, J. B. , & Di Cera, E. (2003). Three‐dimensional models of proteases involved in patterning of the *Drosophila* embryo. Crucial role of predicted cation binding sites. The Journal of Biological Chemistry, 278(13), 11320–11330. 12493753 10.1074/jbc.M211820200

[ece34573-bib-0055] Rosengaus, R. B. , Maxmen, A. B. , Coates, L. E. , & Traniello, J. F. (1998). Disease resistance: A benefit of sociality in the dampwood termite *Zootermopsis angusticollis* (Isoptera: Termopsidae). Behavioral Ecology and Sociobiology, 44(2), 125–134. 10.1007/s002650050523

[ece34573-bib-0056] Rothenbuhler, W. C. (1964). Behavior genetics of nest cleaning in honey bees. IV. Responses of F1 and backcross generations to disease‐killed brood. American Zoologist, 4, 111–123. 14172721 10.1093/icb/4.2.111

[ece34573-bib-0057] Schmid‐Hempel, P. (1998). Parasites in social insects. Princeton NJ: Princeton University Press.

[ece34573-bib-0058] Shi, M. , Lin, X. , Tian, J. , Chen, L. , Chen, X. , Li, C. , … Zhang, Y. Z. (2016). Redefining the invertebrate RNA virosphere. Nature, 540(7634), 539–543. 10.1038/nature20167 27880757

[ece34573-bib-0059] Shokal, U. , Kopydlowski, H. , & Eleftherianos, I. (2017). The distinct function of *Tep2* and *Tep6* in the immune defense of *Drosophila melanogaster* against the pathogen *Photorhabdus* . Virulence, 8(8), 1668–1682. 28498729 10.1080/21505594.2017.1330240 PMC5810505

[ece34573-bib-0060] Simola, D. F. , Wissler, L. , Donahue, G. , Waterhouse, R. M. , Helmkampf, M. , Roux, J. , … Gadau, J. (2013). Social insect genomes exhibit dramatic evolution in gene composition and regulation while preserving regulatory features linked to sociality. Genome Research, 23(8), 1235–1247. 10.1101/gr.155408.113 23636946 PMC3730098

[ece34573-bib-0061] Simone, M. , Evans, J. D. , & Spivak, M. (2009). Resin collection and social immunity in honey bees. Evolution, 63(11), 3016–3022. 10.1111/j.1558-5646.2009.00772.x 19619221

[ece34573-bib-0062] Smith, C. D. , Zimin, A. , Holt, C. , Abouheif, E. , Benton, R. , Cash, E. , … Tsutsui, N. D. (2011). Draft genome of the globally widespread and invasive Argentine ant (*Linepithema humile*). Proceedings of the National Academy of Sciences, 108, 5673–5678. 10.1073/pnas.1008617108 PMC3078359 21282631

[ece34573-bib-0063] Theis, F. J. , Ugelvig, L. V. , Marr, C. , & Cremer, S. (2015). Opposing effects of allogrooming on disease transmission in ant societies. Philosophical Transactions of the Royal Society of London. Series B, Biological Sciences, 370(1669), 20140108 10.1098/rstb.2014.0108 25870394 PMC4410374

[ece34573-bib-0064] Tragust, S. , Mitteregger, B. , Barone, V. , Konrad, M. , Ugelvig, L. V. , & Cremer, S. (2013). Ants disinfect fungus‐exposed brood by oral uptake and spread of their poison. Current Biology, 23(1), 76–82. 10.1016/j.cub.2012.11.034 23246409

[ece34573-bib-0065] Tragust, S. , Ugelvig, L. V. , Chapuisat, M. , Heinze, J. , & Cremer, S. (2013). Pupal cocoons affect sanitary brood care and limit fungal infections in ant colonies. BMC Evolutionary Biology, 13, 225 10.1186/1471-2148-13-225 24125481 PMC3854126

[ece34573-bib-0068] Ugelvig, L. V. , Kronauer, D. J. C. , Schrempf, A. , Heinze, J. , & Cremer, S. (2010). Rapid anti‐pathogen response in ant societies relies on high genetic diversity. Proceedings of the Royal Society B: Biological Sciences, 277, 2821–2828. 10.1098/rspb.2010.0644 20444720 PMC2981995

[ece34573-bib-0069] Viljakainen, L. (2015). Evolutionary genetics of insect innate immunity. Briefings in Functional Genomics, 14, 407–412. 10.1093/bfgp/elv002 25750410 PMC4652032

[ece34573-bib-0070] Viljakainen, L. , Evans, J. D. , Hasselmann, M. , Rueppell, O. , Tingek, S. , & Pamilo, P. (2009). Rapid evolution of immune proteins in social insects. Molecular Biology and Evolution, 26(8), 1791–1801. 10.1093/molbev/msp086 19387012

[ece34573-bib-0071] Viljakainen, L. , Holmberg, I. , Abril, S. , & Jurvansuu, J. (2018). Viruses of invasive Argentine ants from the European Main supercolony: Characterization, interactions and evolution. Journal of General Virology, 99, 1129–1140. 10.1099/jgv.0.001104 29939128

